# Sociological modeling of smart city with the implementation of UN sustainable development goals

**DOI:** 10.1007/s11625-020-00889-5

**Published:** 2021-01-03

**Authors:** Olga Kolesnichenko, Lev Mazelis, Alexander Sotnik, Dariya Yakovleva, Sergey Amelkin, Ivan Grigorevsky, Yuriy Kolesnichenko

**Affiliations:** 1 I. M. Sechenov First Moscow State Medical University, 11/2 Rossolimo Street, 119021 Moscow, Russia; 2 Department of Mathematics and Modeling, Vladivostok State University of Economics and Service, 41 Gogolya Street, 690014 Vladivostok, Russia; 3 ZAO (CJSC) Firm CV PROTEK, 2 Chermyanskaya Street, 2, 127282 Moscow, Russia; 4 Moscow State Linguistic University, 38 Ostozhenka Street, 119034 Moscow, Russia; 5 A.K. Aylamazyan Program Systems Institute of RAS, 4A Peter I Street, 152024 Pereslavl-Zalessky, Russia; 6 Uzgraph, 125195 Moscow, Russia

**Keywords:** API-sociology, Smart and healthy city, Logical artificial intelligence, Community wellness, Sustainable development goals, Sociology of smart city

## Abstract

The COVID-19 pandemic before mass vaccination can be restrained only by the limitation of contacts between people, which makes the digital economy a key condition for survival. More than half of the world’s population lives in urban areas, and many cities have already transformed into “smart” digital/virtual hubs. Digital services ensure city life safe without an economy lockout and unemployment. Urban society strives to be safe, sustainable, well-being, and healthy. We set the task to construct a hybrid sociological and technological concept of a smart city with matched solutions, complementary to each other. Our modeling with the elaborated digital architectures and with the bionic solution for ensuring sufficient data governance showed that a smart city in comparison with the traditional city is tightly interconnected inside like a social “organism”. Society has entered a decisive decade during which the world will change by moving closer towards SDGs targets 2030 as well as by the transformation of cities and their digital infrastructures. It is important to recognize the large vector of sociological transformation as smart cities are just a transition phase to human-centered personal space or smart home. The “atomization” of the world urban population raises the gap problem in achieving SDGs because of different approaches to constructing digital architectures for smart cities or smart homes in countries. The strategy of creating smart cities should bring each citizen closer to SDGs at the individual level, laying in the personal space the principles of sustainable development and wellness of personality.

**Supplementary Information:**

The online version contains supplementary material available at 10.1007/s11625-020-00889-5.

## Introduction: the era of smart cities

The ongoing COVID-19 pandemic, as well as any new lethal pandemic, before mass vaccination can be restrained only by the limitation of contacts between people and keeping social distancing, which makes the digital economy with online services a key condition for survival. Many cities have already transformed into “smart” digital/virtual hubs in which the most of services are provided online with limitations of citizen contacts (PricewaterhouseCoopers 2016; McClellan et al. 2017; Smart City Index 2020; Smart Cities World City Profiles 2020). Smart city approaches have been elaborated to face health care challenges (Islam et al. 2015; Baker et al. 2017; Marjani et al. 2017; Cook et al. 2018; Allen et al. 2019; Akyildiz et al. 2020) and to resist pandemic outbreak expansion with accents on data (Costa and Peixoto 2020; Webb and Toh 2020), social distancing (Cecilia et al. 2020; Nguyen et al. 2020a, b), Internet of Things (IoT) and Artificial Intelligence (AI) (Chamola et al. 2020; Tropea and De Rango 2020), smart health care devices (Jaiswal et al. 2020), and waste management (Onoda 2020). Data governance and digital services ensure city life safe without an economy lockout and unemployment. Around 55% of the world’s population lives in urban areas, and according to the United Nations (UN) urban population will rise up to 68% by 2050 (United Nations Department of Economic and Social Affairs 2018). Thus more than half of the world population will be cover by “smart” cybernetics online governance of their daily life. The “father” of cybernetics Norbert Wiener in 1948 was the first who emphasized that future society governance in the technological world must be based on humanistic values (2000). Such a new phenomenon in the life of society as smart cities poses new challenges to sociology and must give new opportunities to solve modern problems, boost humanistic values, and withstand a wide range of threats.

The future of urban sociology was comprehensively discussed at the joint session of the British and American Sociological Associations in 2001. It was declared that urban sociology faces a variety of challenges with the expansion of numerous issues and themes (Perry and Harding 2002). From the scattered topics investigated within urban sociology, scientific discourse is more and more shifting to the understanding of the interrelated integrity of the urban community. Sociologists need to treat the city as an autonomous social unit, and the better definition is the sociology of the city (Wu 2016). “Urban social” as a sociological category includes globalization, trans‐local and non-human technological influences (Amin 2007), as well as social trust, economic equality and equality of opportunity, fight against corruption, education, health care, and labor market (Rothstein and Uslaner 2005). It is extremely important today to develop a sociological theoretical approach to the creation of smart cities’ theoretical concepts based on a deep and comprehensive understanding of the tasks and needs of the urban population. This scientific task concerns the embracement of “urban social” and frames it into the digital architecture of a smart city.

In essence, a smart city concept is about improving citizens’ lives (Thompson 2016; World Economic Forum 2018). A smart city means Big Data collection and data governance with the IoT and AI. Digital technologies must be the assets in addressing the UN Sustainable Development Goals (SDGs) (United Nations General Assembly 2015; National Academies of Sciences, Engineering, and Medicine 2016) that include ensuring healthy lives and promote well-being for society. The soft city, IT urban infrastructure, city dashboards, urban cybernetics, city digital twin, Internet of Health, smart health care community are the definitions which reflect monitoring of society data flows to make urban society safe, sustainable, well-being, and healthy.

World Health Organization’s Belfast Charter for Healthy Cities declared the values for urban society: equity, human rights, sustainable economic development, and right to health (2018). Despite the significant progress that has been made in health care at the global level, there is still the dangerous risk of emerging following: lethal pandemics including COVID-19, a lot of serious diseases, growing harmful risk factors, and various causes of death, which even according to the optimistic forecast will highly increase the years of life lost in 2040 and beyond (Foreman et al. 2018). Technologies have already transformed health care, breaking down the barriers (clinics walls) and turning medicine into a network of ubiquitous social communications with the new opportunity to decline morbidity and mortality rate, as well as allowing to provide medical care during pandemic via telehealth services without visiting clinics. AI and IoT make it possible to create a digital architecture of a city to maintain health at the population level.

A smart city is built on smart spaces and smart platforms that are ubiquitous and mobile (McClellan et al. 2017; Balletto et al. 2018). Big Data is accumulating in real-time mode. One of the problems is data governance, how to overcome the combinatorial explosion and create such a framework, which will solve the task of data flow routing with massive parallelization and coverage of a city population by a smart network. But the more important problem is how to use technology for improving urban community life while the health issues come to the fore. In this study, we provide some of the comprehensive approaches to modeling complex digital architecture for a smart and healthy city taking into account the needs of people, the urban social characteristics, and the complexity of social governing information flows. A smart city is a new and broad concept with 90% of sociology and only 10% of infrastructure issues, that require new approaches, planning, and engagement of the city community at large (Sandel 2017). We asked ourselves whether it is possible to construct a hybrid sociological and technological concept of a smart city with matched sociological and technological solutions, complementary to each other, which would be a holistic sociomic framework for society and will provide a new opportunity for social development.

## Study design, materials, and methods: the way to embrace social values

**Fig. 1:**
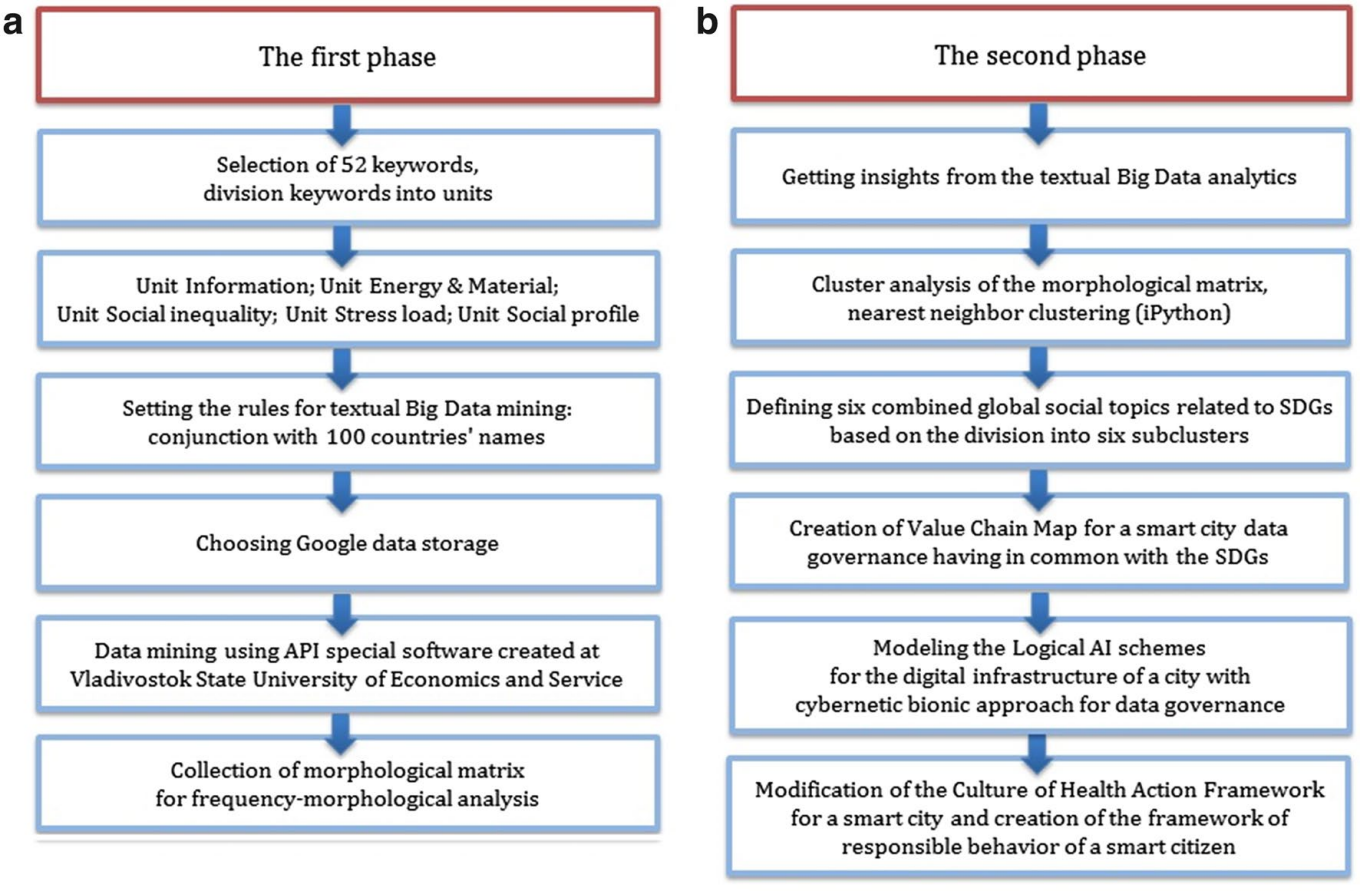
The general scheme of the study

This study is original and based on the investigation of the new trends of the digital age. The study has had two phases. *The first phase* was aimed at API-sociology that is the textual Big Data analytics using API-access to Google texts global data storage. The morphological matrix of several keywords was collected. *The second phase* was dedicated to getting insights from the textual Big Data analytics and modeling Value Chain Map and Logical AI schemes for a smart and healthy city that is the contribution to the emerged sociological theory of smart urban community life. The general scheme of the study is shown in Fig. 1a, b.

During *the first phase* of this study, we used API-sociology as a modern trend in sociology, showing the possibilities of the Internet and computational sociolinguistics for deep sociological investigation. We chose the API-sociology method as the most appropriate one for a large amount of digital research data. The Internet reflects human communication around the world; generated by people texts represent information exchange and opinions. The Google textual data storage is the digital textual language resource for investigation by computational linguistics on the one hand (Michel et al. 2011; Aiden and Michel 2013), and Internet texts are the social data for sociological investigation on the other hand (Lazer et al 2009). We had to choose a large-scale data-driven method in an emerging multidisciplinary field of three coordinates: computation, linguistics, and sociology (Table 1). From the sociolinguistics point of view, textual resources can be seen as a data type that is signaling all kinds of social phenomena (Nguyen et al. 2016). For sociology, which is undergoing the paradigm shift towards data-driven discovery, the level “at the edge” means the possibility to create cross-cutting research questions and resolve complex problems. Sociological analysis of textual Big Data from the Internet is free from such distorting influence as well known Hawthorne effect (Landsberger 1958; Franke and Kaul 1978). Corpus of texts can show the linguistic signs of the new technologies diffusion into society. The phenomenon of increasing diffusion of emerging technologies into society was described by globalist Hirooka (2006). API-sociology combines computational linguistics approach with computational social science as well as sociology field research (Google gathers textual data from an original environment around the world), helping us qualitative estimate social dynamics using quantitative computational linguistics (Kolesnichenko et al. 2016a, b, 2017; Mazelis et al. 2018; Yakovleva et al. 2019).

**Table 1:** Features of investigation methods in an emerging multidisciplinary field of three coordinates: computation, linguistics, and sociology for textual Big Data

Investigation type	Features
Computational linguistics	Machine learning, Natural Language Processing, fact extraction, text mining, information retrieval, n-grams classification, culturomics
Computational sociolinguistics	Allows to study social dynamics in language, studying the language in social contexts (social media data)
Sociology, qualitative and quantitative content analysis	The method is appropriate as part of computational social science: parsing and coding documents to extract information from data, coding categories
Sociology, survey, interview	Digital questionnaire, crowdsourcing, mass digital surveys using Google and other platforms, and social networks or messengers
Sociology, experiment, participant observation, case study, field research	Testing of a hypothesis or to immerse in a group or social setting in order to make observations from an insider perspective. The method is appropriate as part of computational social science, especially social network analysis
Computational social science	Social network analysis is the most used. Social interaction, communication dynamics. Clickstream analysis, sentiment analysis (also known as opinion mining), graph theory, modeling
API-sociology	Large-scale data-driven method. Morphological n-grams frequency analysis of textual Big Data storage using API with the opportunity of time and location tagging data, getting awareness about the situation and dynamics in society. Data-driven sociological analysis of textual Big Data

The global Internet environment is some kind of global audience discussion about various themes. Google captures all sorts of Internet texts: news, articles, advertising, analytics, blogs, and comments. Analyzing textual arrays from the Internet we study the global society and global agenda. We can make an assessment of global society's reflection in information systems. Many keywords about many issues can be investigated. Internet reflects different complex processes and activities from every human to local countries’ issues and global world issues. API-sociology is aimed to find the hidden patterns and meanings in the frequency distribution of selected keywords. API-sociology uses a Big Data approach, which assumes that the collected data isn’t absolutely perfect. The number of counted words may vary over time due to the variability of the Internet itself. But such aggregate measurement allows all variability to be drawn into the investigated general trend. Based on years of studying the Internet including understanding the results of cluster analysis of collected different morphological matrices (Kolesnichenko et al. 2016a, b, 2017; Mazelis et al. 2018; Yakovleva et al. 2019) as well as leaning on existing scientific works (Oliver et al. 1985; Rheingold 2000; Castellano et al. 2009; Gómez et al. 2013; Bode et al. 2014; Dover and Kelman 2018), we formulated the three levels of global society behavior in the Internet space which should be taken into account. Level “Activity”—active presence on the Internet that is different for different countries and depends on the number of computers in the country, literacy, IT education, number of IP connections, Internet accessibility, number of Internet services, amount of country’s population, the intensity of discussion in society, citing of the country on the Internet. Countries' activity on the Internet was investigated by k-means cluster analysis (see in the Supplementary Information, S.I.—Table 1). Level “Knowledge”— different countries have different features of the technological revolution that are reflected in the Internet’s open resources. People write about those technological and economic processes, which have a place in their life, business, country’s agenda. Level “Involvement”—reaction to stress, people write more about those problems which concern them more. Because of differences related to these three levels, the preferable way to analyze data is the cluster analysis within the selected groups of keywords.

Words have become data that can be analyzed to obtain new knowledge and awareness about the situation. There are some successful examples of extraction knowledge through Google textual Big Data analytics: Google Flu project, Google Books Ngram Viewer, Google Trends. In our study, we extracted data from Google using API special software, which has been created at Vladivostok State University of Economics and Service. Selected keywords were counted in a million in conjunction with 100 countries’ names. Data collection (data mining) was implemented during the 2016–2018 years that is now of unique value because there was no unforeseen distorting global pandemic impact on global society that makes a reflection on the Internet, thus we can estimate society under normal conditions to modeling the processes of usual life in smart cities. This is the frequency-morphological analysis of open texts. The morphological matrix of 52 keywords was collected for countries in the English language (see in the Supplementary Information, S.I.—Table 2). Our study has been going on for 6 years, and we have gradually chosen keywords, which can reflect in total the processes taking place in society and in the economy.

## Results: from the morphological matrix to Value Chain Map

**Fig. 2:**
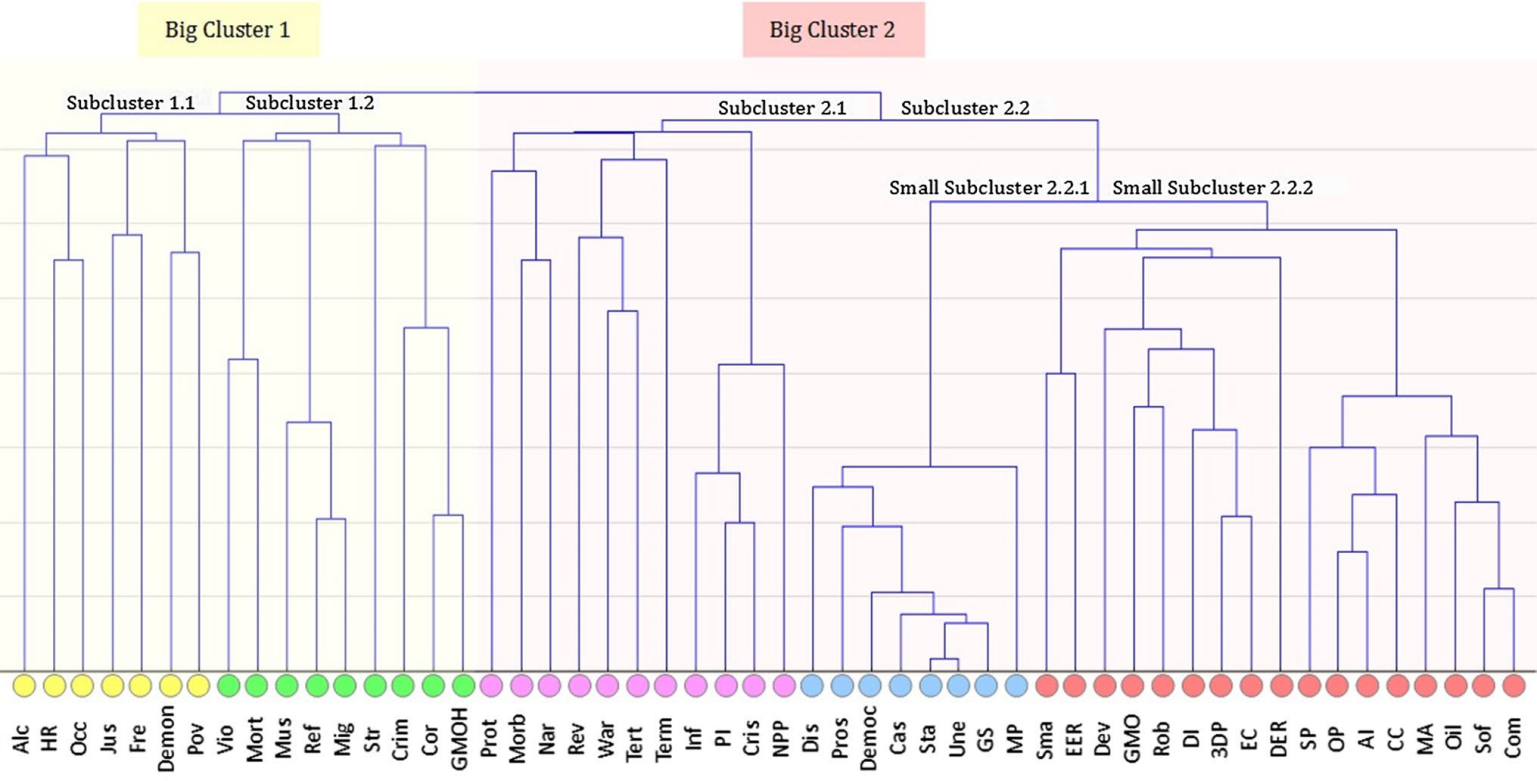
The dendrogram of the morphological matrix, 52 keywords, and 100 countries; analyzed parameters – keywords (Google API). Description of words abbreviations (words were subjected to mathematical processing without abbreviations). Subcluster 1.1: *Alc* alcoholism, *HR* human rights, *Occ* occupation, *Jus* justice, *Fre* freedom, *Demon* demonstration, *Pov* poverty. Subcluster 1.2: *Vio* violation, *Mort* mortality, *Mus* Muslims, *Ref* refugees, *Mig* migrants, *Str* strike, *Crim* crime, *Cor* corruption, *GMOH* GMO harmful. Subcluster 2.1: *Prot* protest, *Morb* morbidity, *Nar* narcotic, *Rev* revolution, *War* war, *Tert* terrorist, *Term* terrorism, *Inf* inflation, *PI* price increase, *Cris* crisis, *NPP* nuclear power plant. Subcluster 2.2.1: *Dis* dismissal, *Pros* prosperity, *Democ* democracy, *Cas* casualties, *Sta* stability, *Une* unemployment, *GS* gas supplies, *MP* mobile phone. Subcluster 2.2.2: *Sma* smartphone, *EER* euro exchange rate, *Dev* development, *GMO* GMO, *Rob* robotics, *DI* drip irrigation, *3DP* 3D printing, *EC* electric cars, *DER* dollar exchange rate, *SP* solar panel, *OP* oil price, *AI* artificial intelligence, *CC* cloud computing, *MA* mobile app, *Oil* Oil, *Sof* software, *Com* computer

The morphological matrix was analyzed by cluster analysis (Fig. 2). The single-linkage clustering (nearest neighbor clustering) was implemented in iPython (NumPy, Pandas, and Sklearn). This quantitative technique classifies data into homogeneous subgroups. The vertical axis of the dendrogram represents the distance or dissimilarity between clusters. The horizontal axis represents the objects (keywords, its frequency) and clusters. The dendrogram shows the two big clusters and six subclusters. Based on the keywords division into subclusters the six combined global social topics were defined which are important for society.

Big cluster 1: poverty, mortality, and corruption.

- *Subcluster 1.1* “Free enterprise and human rights to live without poverty”
  Main keywords grouped into a subcluster: *human rights*, *demonstration*, *freedom*, *justice*, *poverty*.
  This reflects the poverty and related protest activity.
- *Subcluster 1.2. “Mortality in the aspect of crime”. *
  Main keywords grouped into subcluster: *mortality*, *violation*, *crime*.
  This reflects deaths from criminals.
  “Fighting corruption for ensuring food security”
  Main keywords grouped into subcluster: *corruption*, *GMO harmful*.
  This reflects corruption cases related to label genetically modified foods and phobia about the harmful effects of GMO (see in the Supplementary Information, S.I.–Fig. 1 the countries’ names in conjunction with which we found the percentage predominance of keywords *GMO harmful* over just *GMO* on the Internet).

Big cluster 2: democracy, health, and development.

- *Subcluster 2.1* “Health as a personal safety issue”
  Main keywords grouped into subcluster: morbidity, narcotic, crisis, inflation, price increase, terrorism, war.
  This reflects the health problem not only during usual life, including the growing prevalence of drug addiction but with an accent of war and terrorism consequences.
- *Subcluster 2.2.1* “Democracy as the condition for prosperity”
  Main keywords grouped into subcluster: *democracy*, *prosperity*, *stability*, *unemployment*.
  This reflects the democratic way of economic development.
- *Subcluster 2.2.2* “Electricity availability with containment of price growth”
  Main keywords grouped into subcluster: *dollar exchange rate*, *euro exchange rate*, *solar panel*, *development*.
  This reflects the growing need for electricity for new digital technologies, green energy, and energy availability issues.

**Fig. 3:**
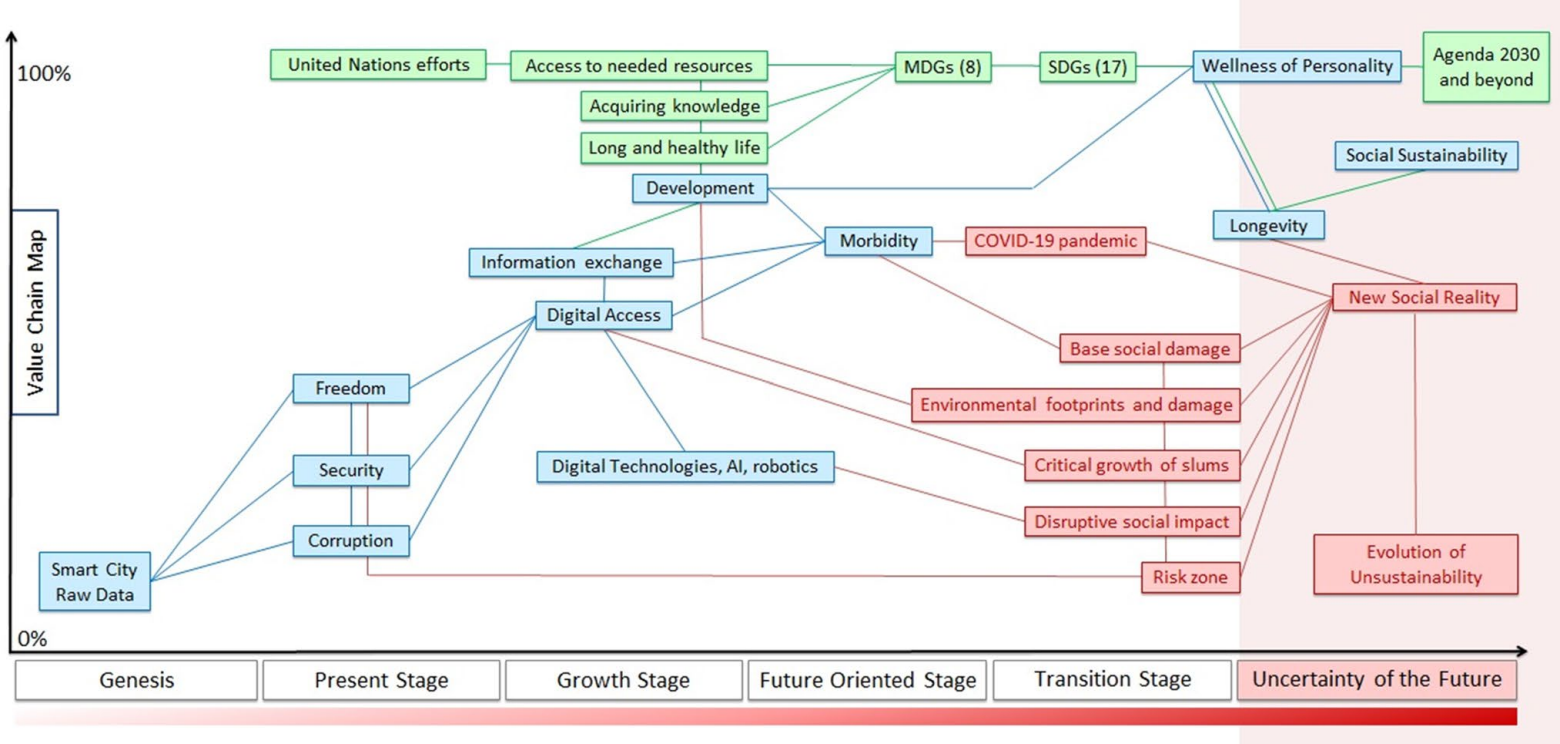
The Value Chain Map for results of text Big Data analytics (Google API) related to Sustainable Development Goals. Blue boxes and lines—the chain based on revealed by API-sociology combined global social topics. Green boxes and lines—the reflection of the global approach of the United Nations for sustainable development. Red boxes and lines—the worst scenarios, threats, and challenges. *MDGs* Millennium Development Goals, *SDGs* Sustainable Development Goals

**Fig. 4:**
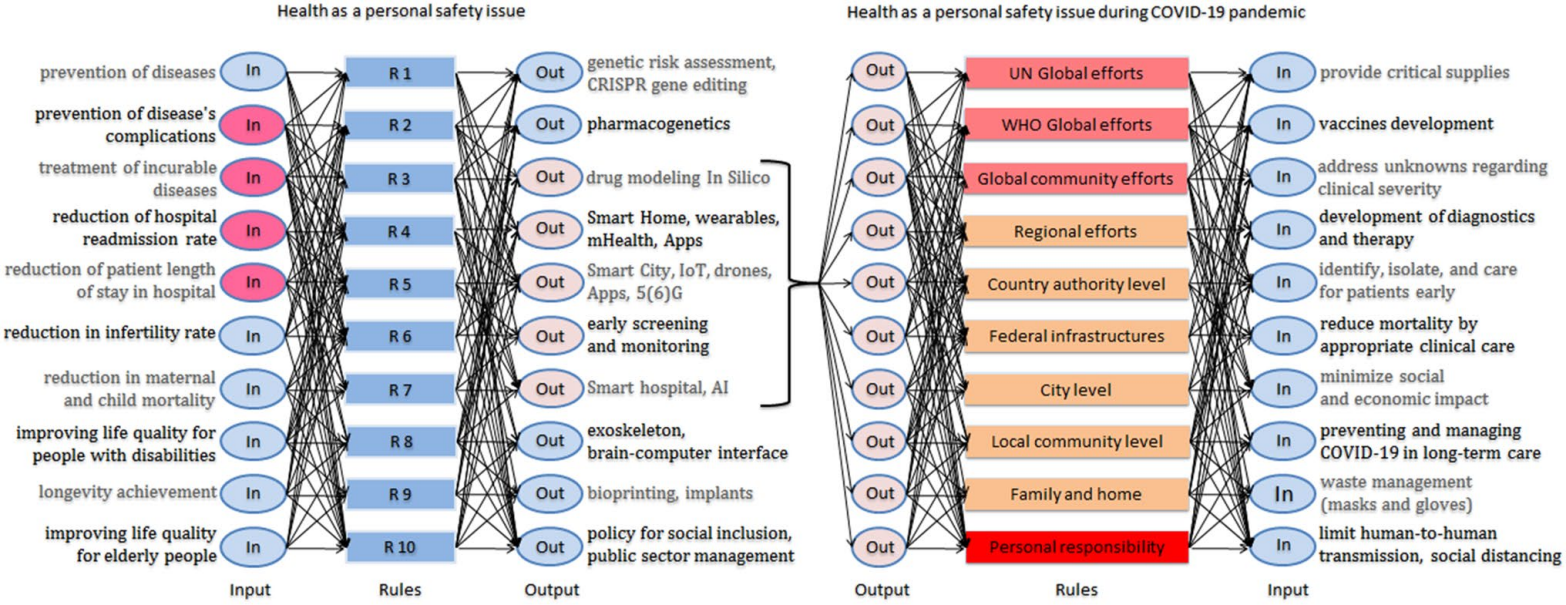
Logical AI scheme “Health as a personal safety issue” for a smart city with showing the influence of COVID-19 pandemic (the COVID-related branch)

**Fig. 5:**
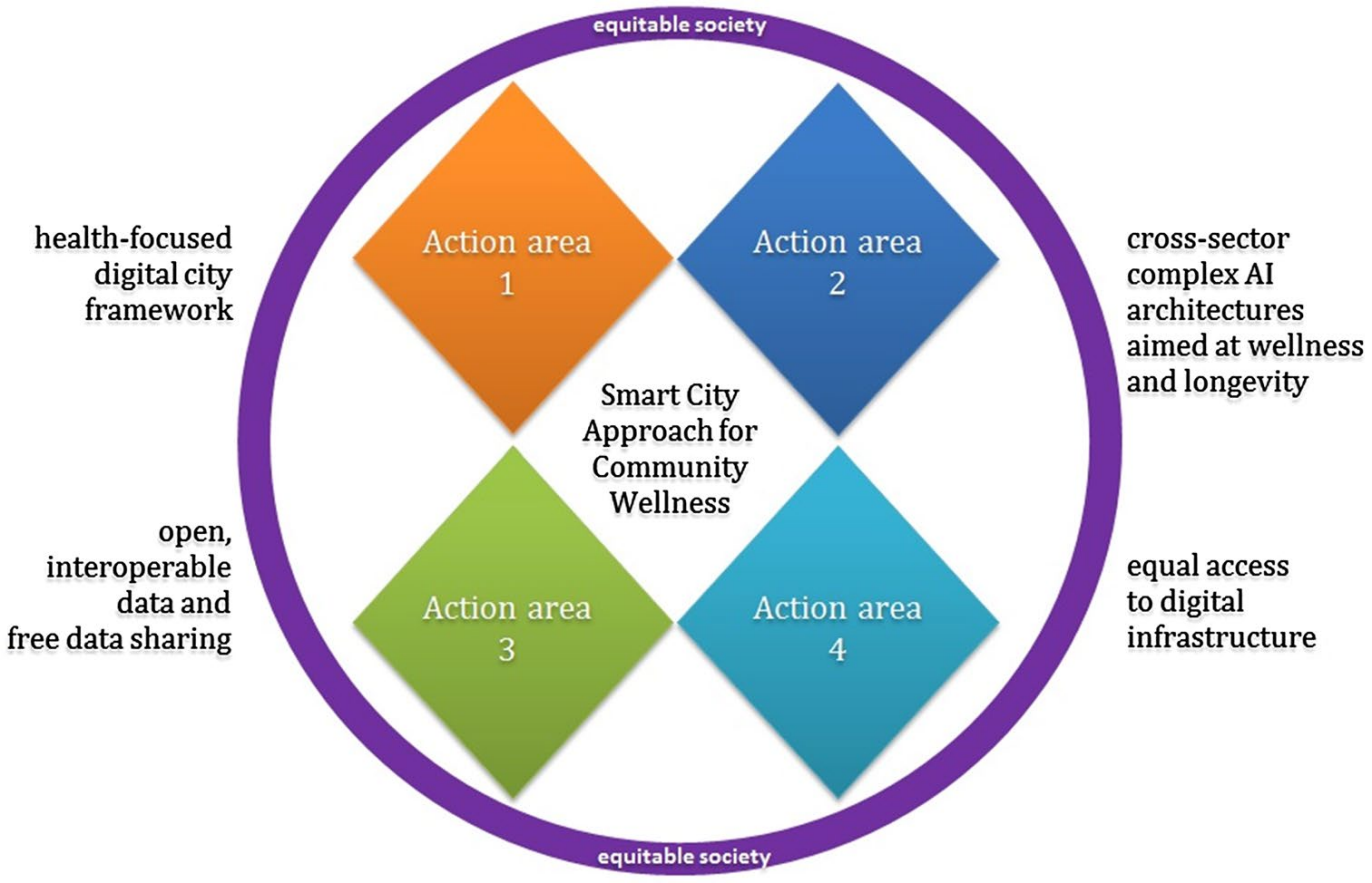
Smart City Approach for Community Wellness – Culture of Health Action Framework adapted for Smart City. Primary Culture of Health Action Framework was developed by Robert Wood Johnson Foundation & RAND Corporation (Acosta et al. 2016; Plough et al. 2018)

The revealed by API-sociology combined global social topics have in common with the SDGs (Table 2), which are the result of a complex worldwide negotiation process and reflect the needs of people around the world. Our findings confirm the ability of API-sociology to identify signs of the true discussion in society, as well as points out the importance of themes related to SDGs for people. We ranked six combined global social topics from first to last depending on the intensity of the keywords’ frequency mentioned in our API-sociology investigation (see additional diagrams that show the difference of keywords frequency in the Supplementary Information, S.I.–Figs. 2-5) and created the Value Chain Map which has synergistic incorporated SDGs (Fig. 3).

**Table 2:** Matching of API-Sociology insights (combined global social topics) with UN Sustainable Development Goals

Combined global social topics^a^, API-Sociology	UN Sustainable Development Goals^b^
1. Free enterprise and human rights to live without poverty	Goal 1. End poverty in all its forms everywhere Goal 8. Promote sustained, inclusive and sustainable economic growth, full and productive employment and decent work for all Goal 9. Build resilient infrastructure, promote inclusive and sustainable industrialization and foster innovation Goal 12. Ensure sustainable consumption and production patterns
2. Health as a personal safety issue	Goal 3. Ensure healthy lives and promote well-being for all at all ages
3. Electricity availability with containment of price growth	Goal 7 Ensure access to affordable, reliable, sustainable and modern energy for all
4. Democracy as the condition for prosperity	Goal 4. Ensure inclusive and equitable quality education and promote lifelong learning opportunities for all Goal 5. Achieve gender equality and empower all women and girls Goal 10. Reduce inequality within and among countries Goal 16. Promote peaceful and inclusive societies for sustainable development, provide access to justice for all and build effective, accountable and inclusive institutions at all levels
5. Mortality in the aspect of crime	Goal 11. Make cities and human settlements inclusive, safe, resilient and sustainable
6. Fighting corruption for ensuring food security	Goal 2. End hunger, achieve food security and improved nutrition and promote sustainable agriculture Goal 6. Ensure availability and sustainable management of water and sanitation for all

^a^Ecology and humaneness to nature/animals weren’t considered in this study despite the importance of these issues and disruptive influencing to the natural environment of all mention topics. These issues require a deep and large separate study by API-Sociology

^b^Goals 13, 14, 15, 17 aren’t mentioned

During *the second phase* of this study, we considered Value Chain Map as an interrelated chain of growth drivers for modern social reality in that people are beginning to live in smart cities with digital data governance. We were inspired by the “Mapping of a Strategy” method invented by Wardley (2014, 2015, 2019). The key positions are distributed on the map in relation to the stages of the strategic evolution in time. Stage “Genesis” is as root for governance, the key point is raw data collected from smart city systems around the whole social life. Raw data first passes through three filters which determine the way of data usage. The fight is observed worldwide between open source and secure digital data. There is a confrontation with three forces at the “Present stage”: respect for freedom, ensuring security, and corruption penetration. Bad equilibrium with data closure and corruption increases risks of data governance fails that will make citizens’ well-being worse and leads to trust loss within society.

Data per se has less value than digital solutions and services created due to data. Digital access for people determines the quality of life; violation of access to the Internet is equal to a violation of human rights (United Nations General Assembly 2011, 2016). Slow adoption of smart city technologies will contribute to the growth of the slum population. Digital rights as well as digital technologies, especially AI and robotics are the base features of the “Growth stage” and development. But at the same time, AI and robotics have a strengthening disruptive effect on society, contributing to the growth of unemployment in the production sectors of the outgoing economic order of the past. Together a pronounced social impact by technologies with risks of corruption and data hiding will lead to the formation of a new negative social reality. Nowadays the formation of data governance rules for the smart city concept determines future risks.

Following the revealed high frequency of mention of keyword *morbidity* in our API-sociology investigation (see in the Supplementary Information, S.I.–Figs. 2, 4), we put the issue of morbidity ahead of the development as the main task for “Future oriented stage”. The high frequency of mention of *morbidity* reflects the huge interest of the global Internet audience to this theme even before the appearance of COVID-19 pandemic. This correlates with the continuous growth of the global burden of diseases (Foreman et al. 2018), which determines base social damage with the negative effect on the development. Thus despite that economic development is a source of well-being we placed health issues at the more important place on Value Chain Map. The current transition to the digital economy carries with it risks to humanity which hasn’t been in history before. Care about people should be at the center of digital progress. According to the World Economic Forum report alongside with demand for digital technology specialists, the greatest growth sector in the job market is care roles that transform economy more into “care economy” (Ratcheva et al. 2020). Special attention should be paid to digital medicine as it dissolves in the Internet of Things. Medicine is becoming an essential part of the digital economy where health (wellness) is a resource for economic growth. Digital medicine provides a personalized ubiquitous approach and care, and we can consider the wellness of personality instead of the health of depersonalized statistical humans. The main feature of the future will be the reaching of a longer life. During the next 20 years, life expectancy will increase up to 90 years (Foreman et al. 2018). Longevity can be denoted as the goal of the current cybernetic revolution.

Development is always interlinked with resource consumption, waste growth, negative impact on climate and nature. The “Transition stage” included the worst scenarios, threats, and challenges accompanying the designated points of the strategy. At the top of the scheme are the UN’s evolutionary efforts aimed at ensuring transit to the future through the best path, avoiding the worst-case scenarios. In contrast to the chain built on the basis of our textual analytics of the global Internet discussion with an emphasis on overcoming the morbidity growth, the UN efforts have shifted the focus from the isolated solution of health problems (Millennium Development Goals had 3 health goals of 8, and SDGs have 1 of 17) to the comprehensive approach. Today UN implements the omnipresent strategy which is in best relevancy to solving the health tasks because all interlinked social, economic, and environmental issues are synergistically taken into account.

The presentation of the stage “Uncertainty of the Future” is the goal for the strategy mapping. We can see what confronts social sustainability achievement, from what trends the obstacles and challenges come, from what features might consist of possible negative future scenarios. Talking about the global threats to sustainable development the world expected that AI would become so advanced that humanity undergoes a dramatic and irreversible change (Hawking et al. 2017) or in NATO’s report “Multiple Futures Project: Navigating Towards 2030” were described four scenarios revealing the most dangerous emerging security challenges (NATO Allied Command Transformation 2009), but first the current COVID-19 pandemic is turning into the scenario of the singularity of biological threats. Wellness of personality has the highest value in the presented Value Chain Map. Wellness from the perspective of Agenda 2030 can be reached through rights, opportunity, and responsibility of personality (of every citizen). Proposed by us the map helps to orient before making the decision. Incorporation of the textual Big Data analytics of the global Internet into the map allows making the agile assessment of SDGs reflection in people global discussion and provides a fast reaction to any change of accents. It is important to emphasize that due to the presented Value Chain Map we manage to show how to build a sustainable development strategy for the smart city from the root, from raw data collection to successful data governance and implementation of SDGs.

## Modeling of smart and healthy city

**Fig. 6:**
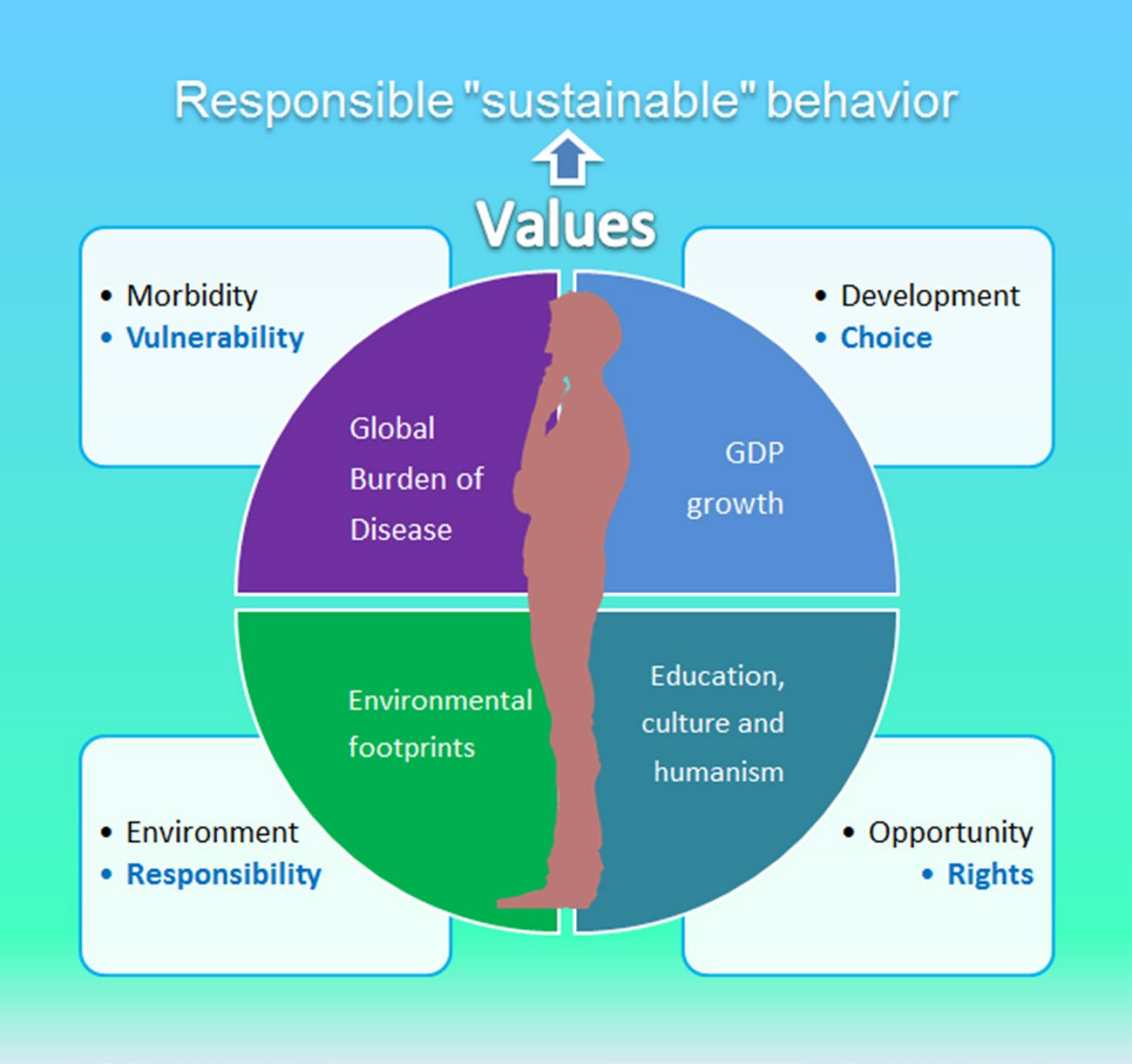
The framework of responsible behavior of the citizen of a smart city in the context of SDGs achieving

Value Chain Map based on findings related to SDGs was applied to the strategy of the digital economy of a smart city. In this way, we modeled Logical AI schemes, which are designed to data governance the digital infrastructure of a city of the future, which will be a place for healthy life and longevity. We presented examples of four schemes that can be called architectures for smart city data governance (Tables 3, 4, 5 and 6; also see in the Supplementary Information, S.I.—Figs. 6, 7, 8, 9, 10, 11 and 12). The architectures reflect the hybrid matched sociological and technological solutions. Parameters for Input include the most important challenges that must be addressed to ensure the well-being of society. Parameters for Output contain the new technologies and approaches for solving the selected problems. Some of the technologies are just at the beginning of implementation or discussed in the various Internet resources as emerging technologies. The digital design of a smart city should be focused on the most modern and future technologies.

**Table 3:** Architecture “Health as a personal safety issue” for smart city data governance

Parameters for input	Parameters FOR Output
Prevention of diseases	Genetic risk assessment, CRISPR gene editing
Prevention of disease’s complications	Pharmacogenetics
Treatment of incurable diseases	Drug modeling In Silico
Reduction of hospital readmission rate	Smart Home, wearables, IoT, mHealth, Apps
Reduction of patient length of stay in hospital	Smart City, IoT, drones, Apps, 5(6)G
Reduction in infertility rate	Early screening and monitoring, electronic health records
Reduction in maternal and child mortality	Smart hospital, AI
Improving life quality for people with disabilities	Wearable devices, exoskeleton, brain–computer interface
Longevity achievement	3D–printing, bioprinting, implant–tissue interface, neuron–like electronics, neurogenesis
Improving life quality for elderly people	Policy for social inclusion, public sector management

Note for Tables 3, 4, 5 and 6: The emerging technologies are described in detail on web resources: World Economic Forum Strategic Intelligence platform (https://intelligence.weforum.org/) and Envisioning Technology Radar platform (https://viz.envisioning.io/wgs-citizenship/)

**Table 4:** Architecture “Free enterprise and human rights to live without poverty” for smart city data governance

Parameters for input	Parameters for output
Simple registration of a new private business	Ecosystem for innovation-driven entrepreneurship
Accelerators and venture funding	New job creation
Tax cut and tax exemption	Agile governance, smart tax, real time individual tax planning
Low loan interest rate	Social impact-driven entrepreneurship
Minimization of administrative checks	Smart contract, administrative intelligence
Law enforcement control	Blockchain
Reduction in spending on infrastructure	Smart City, IoT, Mobile Apps, AI Energy Grid
Employer’s tax incentives for disabled employees	Smart Home, IoT, Mobile Apps
Increasing economic literacy	Digital public policy, urban sensor Web
Gender and age equality and support for pregnant	Corporate social innovation

**Table 5:** Architecture “Democracy as the condition for prosperity” for smart city data governance

Parameters for input	Parameters for output
Overcoming information inequality	Free wireless ubiquitous access to the Internet
Freedom of speech and expression	Encrypting, biometric identification
Common values	Citizen-driven crowdsensing platform, virtual city
Local government	Hybrid Cloud, distributed edge
Open Data	Government Cloud
Equal access to education and skills	Virtual and Augmented Reality, gamification of life
Equal access to medical care and drugs accessibility	Smart City, IoT platforms, AI, Smart Home, mHealth, Mobile App
Mobility and property rights	Virtual world passport, e–residency
Rights to work and retire	Human enhancement, full body avatar
Rights to have family and children	Administrative intelligence for public civil services

**Table 6:** Architecture “Fighting corruption for ensuring food security” for smart city data governance

Parameters for input	Parameters for output
Eradicating hunger and malnutrition	Social impact-driven entrepreneurship
Eradicating food deserts	Smart City, e-commerce, e-retail, marketplace
Fight with obesity, anemia, deficiency of vitamins	Smart Home, IoT, mHealth, Apps, electronic product code
Food pricing control	Citizen-driven crowdsensing platform
GMO regulation and control	Government Cloud, administrative intelligence

The architecture “Health as a personal safety issue” includes basic health care tasks for city community wellness (Fig. 4). We chose the ten tasks based on a general view of a healthcare organization, SDG 3 (https://sdgs.un.org/goals/goal3) as well as WHO Global Health Estimates vision (Global Health Estimates 2020). These ten tasks are oriented to help people with the leading causes of death—ischaemic heart disease, cerebrovascular diseases, chronic obstructive pulmonary diseases, lung cancer, diabetes mellitus, etc. (Mathers and Loncar 2006; Foreman et al. 2018). The determined ten tasks can be solved through several technological directions in different combinations that are shown on the AI scheme. For example, the task “reduction of hospital readmission rate” is important for health care. A patient can be successfully treated at a smart home with mHealth, different Apps, and wearable devices. AI technology in a smart hospital helps to shorten the length of inpatient treatment. No less important than digital technologies is the policy for social inclusion and public sector management. Social management should be changed according to technological progress. The inclusion of all tasks into the unified scheme allows integrating each solution with several options (see in the Supplementary Information, S.I.—Figs. 6, 7, 8 and 9).

The COVID-19 pandemic is a full-blown crisis in itself (Guterres 2020); during the COVID-19 pandemic, the studied landscape of morbidity and causes of mortality of the population has dramatically changed because of the crucial impact of Coronavirus (World Health Organization 2020a). The pandemic has disrupted health care systems (Brunier and Harris 2020; Cutler 2020; Ducarme 2020; Thorlby et al. 2020); the direct impact COVID-19 is having on mortality and cost to the health care both are significant (McKinsey & Company 2020). Since the COVID-19 outbreak, the people who need treatment for diseases like cancer, cardiovascular disease, diabetes, etc. haven’t been receiving the health services and medicines of full value they need; SDG target 3.4 on non-communicable diseases (by 2030, reduce by one-third premature mortality from non-communicable diseases) is off track (Ghebreyesus 2020; World Health Organization 2020a; Gutierrez and Bertozzi 2020). Also, people living with non-communicable diseases are more vulnerable to becoming severely ill or dying from COVID-19 (World Health Organization 2020a). We added to architecture “Health as a personal safety issue” the COVID-related branch (Fig. 4). We show a multi-layered system of response to the epidemic with tasks (as Inputs) defined by the UN and WHO (United Nations Secretary-General 2020; World Health Organization 2020b, c), and the most effective technological solutions (as Outputs). The pandemic has enhanced the need and ability to incorporate technological solutions at a pace never seen before because technologies can provide comprehensive digital remote control, monitoring, care, and diagnosis (Chamola et al. 2020; Clipper 2020; Jamshidi et al. 2020; Pham et al. 2020). The highlighted outputs also should be strengthened for the care of patients with non-communicable diseases (tasks “prevention of disease’s complications”, “treatment of incurable diseases”, “reduction of hospital readmission rate”, “reduction of patient length of stay in hospital”).

The architectures “Free enterprise and human rights to live without poverty”, “Democracy as the condition for prosperity”, and “Fighting corruption for ensuring food security” are shown in Supplementary Information (S.I.—Figs. 10, 11 and 12). These schemes reflect the economy, social rights, and conditions for urban population well-being based on SDGs and tasks of the UN Decade of Action on Nutrition (Rodrigues-Birkett and Branca 2018). In the presented digital architectures, we demonstrated the wide range of applications of new technologies and approaches to comprehensively solve daily basic problems for the urban population. A city’s digital architecture shouldn’t distort basic social processes. Moreover, digital technologies make it possible to take into account the SDGs in data governance, which are demanded by society.

Data governance for a smart city should be based on the framework for massive parallelization to overcome the combinatorial explosion. The cybernetic bionic approach can be implemented in our sociological modeling. The best model is the thalamus—the brain’s structure. Thalamus function is compared with a relay or commutator that redirects all sensory or motor inputs to different areas of the cerebral cortex (Melchitzky and Lewis 2009; Mitchell et al. 2014). In nature, information is redirected by three forms due to functional differences of thalamic nuclei: specific relay nuclei, association relay nuclei, and diffuse-projection nuclei (Melchitzky and Lewis 2009).

The specific relay nuclei process input from a single sensory modality and project it to a specific area of the cerebral cortex. A specific relay way can be extrapolated to data governance as the information flow of personal problems and solutions. The association relay nuclei receive highly processed input from more than one source and project it to larger areas of the cerebral cortex. An association relay way can be extrapolated to analytical information and problem solving for a sector. The diffuse-projection nuclei receive input from diverse sources and project it to widespread areas of the cerebral cortex. A diffuse-projection way can be extrapolated to IoT Big Data flows and sociomic comprehensive regulation (see in the Supplementary Information, S.I.—Fig. 13). Considering that a smart city needs the relevant digital environment with High-Performance Computing, this should be a multiprocessor environment with four components of Flynn’s taxonomy: for specific relay data flow—single instruction and single data; for association relay data flow—multiple instruction and single data, and single instruction and multiple data; for diffuse-projection data flow—multiple instruction and multiple data.

Due to the proposed cybernetic bionic approach, the coverage of the whole city population together with personalized support for each citizen can be achieved by the thalamus-like smart network. Trying to overcome the technological problem related to massive parallelization we had to propose the bionic approach like the “brain” governing the whole “organism”. This fact points out to technological necessity for the smart city to be an autonomous and united social unit. Also, this confirms the accuracy of the modern definition “sociology of the city” with treating the city as an autonomous interrelated social unit. The digital architecture of a smart city can be called a city digital twin. It is very important to understand the holistic nature of a smart city. In fact, while creating digital architectures for cities we should shape the digital twin of the city as a whole social “organism” (see in the Supplementary Information, S.I.–Fig. 14).

## Discussion: health-focused digital city framework

It can definitely be said that the topic of smart cities is now beyond the scope of computer science and becoming an advanced tool for health care and social managing. A smart city is the best investment in public health. The priority of health as a primary asset is considered worldwide for development planning in the following decades (Davies et al. 2018; Kruk et al. 2018). The most fundamental shift affecting global development is the rising burden of disease and mortality (Schäferhoff et al. 2015). It is necessary from the very beginning to put health care priority developing digital architectures of smart cities. For citizens’ community, all problems affecting health can be monitored comprehensively including ecology, nutrition, psychological health, safety, ergonomics, employment, and income. Open, interoperable data and free data sharing (as FHIR API) are the key conditions for digital society wellness. Open IoT platforms with AI (Martins et al. 2020) will drive governance of all processes (as Microsoft CityNext, Watson IoT Platform, NEC Platform—Cloud City Operations Center, Schneider Electric—EcoStruxure™, Philips CityTouch & SAP HANA, Siemens—Digital City Solutions, Compta Smart Cities Platform, and other) including health care (Brack and Castillo 2015; Cygan et al. 2018; Batra et al. 2019). Multilevel architectures of a smart city should have a security system for protection against hacking and failures.

Today pharmacological companies are very active in the development of digital services for citizens. They create digital logistics schemes to purchase the necessary medicines in the city pharmacies network with discounts and delivery, as well as to get the doctors consultations online or in a clinic. There are some other advanced cloud-based projects, for example, Atrium Health and Cerner HealtheIntent^SM^ in the USA. Atrium Health connects citizens with on-demand care and provides the region’s primary care network. Service aggregates different data: DNA sequencing, health monitoring parameters, environmental pollution, and chatbot surveys “Coronavirus Assessment Tool”. Cerner HealtheIntent is the health management platform that enables to aggregate, transform and reconcile data across the continuum of personalized care (citizens’ data from clinics, hospitals, homes, fitness centers, retail pharmacies, workplaces); the platform provides supply and staff resources during COVID-19 outbreak. Such systems are a significant step towards creating a full-fledged digital architecture of a smart and healthy city.

We investigated the topics that were mentioned in the scientific articles about smart cities. We implemented several queries using IEEE Xplore Digital Library API Dynamic Query Tool: smart city; smart healthy city; smart city + artificial intelligence, SDGs, or COVID (see in the Supplementary Information, S.I.—Fig. 15, 16, 17, 18 19 and 20). IEEE library consists of millions of articles written around the world mostly about technologies. The investigation revealed the most mentioned terms with respect to smart cities: data, IoT, communications, architectures, applications, networks, sensors, traffic, wireless technologies, and energy. The articles about smart and healthy cities have more accents on the ecosystems, environment, waste management, air pollution, water quality, and plastic waste of households. Among the worldwide publications about smart and healthy cities also were mentioned mobile technology, elderly citizens, healthy aging, healthy lifestyle, immunization monitoring, emotional wellness, disaster evacuation management, security environment, urban mobility, food safety, healthy products, and green energy. In articles dedicated to smart cities with AI the most mentioned terms were Big Data, machine learning, and electrical energy. The articles about smart cities during the COVID-19 pandemic are more devoted to social distancing, communications, remote technologies, unmanned aerial vehicles, cybersecurity, and waste management. Overall researchers have paid the most attention to the technologies, ecology, and energy of a smart city. But there is insufficient attention to the complex medical aspects and health policy incorporated into the sociology of a city. Articles that included the theme of SDGs have accents on solar or clean energy, clean water, food security, supply chains, quality education, and smart urbanism.

Modern health policy aims to create community-based programs and foster a culture of health in society (Acosta et al. 2016; Teno et al. 2017; Plough et al. 2018). We modified the Culture of Health Action Framework (Fig. 5) keeping the community-based approach firstly elaborated by the Robert Wood Johnson Foundation & RAND Corporation. The modified framework provides the culture of the digital world to construct smart cities to successfully transit to a sustainable future, with the demanded accent on health and wellness. The four action areas include a health-focused digital city framework; cross-sector complex AI architectures aimed at wellness and longevity; open, interoperable data and free data sharing; and equal access to digital infrastructure. These four areas not only embrace what we showed on the Value Chain Map and Logical AI schemes but are placed at a significant place in the digital rights for citizens. Thus due to this framework, we emphasized the crucial importance of community social culture-shaping for digital practice without that the successful transit to a sustainable future is impossible.

Scientists offered different models and frameworks for the implementation of SDGs. In this article, the authors don’t set a task to make a comprehensive review of existing in the world science works devoted to SDGs modeling. But we provide a short review of some proposed models and frameworks from the ScienceDirect and IEEE Xplore databases. Urban planning is associated with major challenges for the implementation of the 2030 Agenda for Sustainable Development and needs the model approach for the assessment of sustainability in a city. Marjaba et al. (2020) elaborated the Sustainability Performance Metric framework for single-family-detached housing; the model includes indicators of ecological impacts, energy consumption, social and health issues, economic data, and whole life cycle estimation. Stokes and Seto (2019) emphasized that urbanization is one of the defining trends of the twenty-first century, shaping the development outcomes integral to human well-being on the one hand, and threaten biodiversity, promoting growing emissions and pollution on the other hand. The authors developed a typology of urbanization (seven distinct classes) with an accent on electrification on a local level, helping identify areas of slums. In the international study led by the European Commission (Vanham et al. 2019) the model of environmental footprint family was proposed for the assessment and quantification of environmental sustainability in relationship with nine planetary boundaries (Rockström et al. 2009), SDGs, and Water-food-energy-ecosystem (WEFE) nexus (Roidt and De Strasser 2018). Requejo-Castro et al. (2020) proposed a data-driven Bayesian network approach to identify SDGs interlinkages across the whole 2030 Agenda with a special focus on SDG 6, related to water and sanitation.

Kørnøv et al. (2020) created the conceptual model with six levels of SDGs and environmental assessment integration. The model shows the new research field related to the decision-making process for the implementation of SDGs in that environmental assessment can be strengthened and sustainability assessment can raise environmental ambition. Waage et al. (2015) discussed the interdependency of the health and wellbeing goals with other goals. The authors highlighted that all SDGs can be grouped into three levels—wellbeing, infrastructure, and natural environment; and health is synergistically linking to all three levels. They compared the Millennium Development Goals which had three concerned health goals of the eight, with one health goal among the enlarged list of SDGs. This evolution of goals showed a growing understanding of what way should be to achieve sustainable development. The proposed framework by Waage et al. shows that without taking into account all SDGs together in a synergistic manner global health will probably be achieved at the expense of natural environment goals until resources are exhausted. Pascual et al. (2017) emphasized that the diversity of values, especially related to nature, and individual self-interested behaviour, associated with a belief in material economic growth, produces diverse and conflicting perspectives in terms of ways of achieving SDGs worldwide. Authors created the model of visualizing the diversity of values around nature and quality of life for the ability to bridging and mobilize transdisciplinary collaboration across a broad range of natural and social fields towards the sustainable pathway. Ramirez-Rubio et al. (2019) following The Helsinki Statement on Health in All Policies (World Health Organization 2013) confirmed that the SDGs provide a holistic and integrated framework to address health-related sustainable development challenges and give an opportunity to implement Health in All Policies approach in the best way. Authors argued that a more comprehensive Health in All Policies vision within the SDGs could be beneficial to improve urban planning for cities worldwide as well as to achieve the 2030 SDG Agenda.

Describing the conceptual model of unsustainability Snyder (2020) stressed that understanding how society transits from an unsustainable to a sustainable state is the most important question regarding environmental problems in the twenty-first century. The author mentioned the Anthropocene’s environmental crisis, a continuum of unsustainability inherent to people’s history, Malthus theory of resource limitation while population growth, and ecosystem engineering has been implemented by humankind. However, Kapitsa (2008) built the hyperbolic model of the global growth of humankind and proved that Malthus resource theory isn’t correct, because the only condition for survival is collective interaction and information exchange. Thus equal access to digital infrastructure, information, and behavior values exchange, and based on SDGs collective interaction through digital platforms should underlay the life of a smart city—this is the model which we propose considering the city as the main result of human ecosystem engineering.

## Conclusion. Transition from city to smart home: can we bring SDGs to every home?

In this study, we make a contribution to scientific discourse about the sociology of the city in a modern context related to “smart” digital technologies and SDGs implementation. We set the task to construct a hybrid sociological and technological concept of a smart city with matched sociological and technological solutions, complementary to each other. The shown AI schemes (architectures) are devoted to finding the solution to this task and they correspond to revealed actual global social topics by our API-sociology approach. Based on revealed social values and also on our view about the importance of health issues for society we described the model of “smart city”. Our modeling with the bionic solution for ensuring sufficient data governance showed that smart city in comparison with the traditional city which doesn’t have the digital infrastructure, as well as with a smart city which has the digital infrastructure but doesn’t have conceptually united governance by all social processes, is tightly interconnected inside like social “organism”.

We have entered a decade during which the world will change by moving closer towards SDGs targets 2030 as well as by the transformation of cities and their digital infrastructures. We can absolutely sure that all new technologies such as IoT, AI, the Internet, mobile cloud, will be used in cities. But without a special strategy, there is no guarantee that in smart cities these technologies will make cities clean, fair, green, sustainable, safe, healthy, affordable, or resilient with higher ethical values of liberty, and equality (Sterling 2018). The UN initiative “United for Smart Sustainable Cities” (U4SSC) coordinated by the International Telecommunication Union (ITU) has facilitated the development of internationally recognized key performance indicators for cities to make infrastructure relevant to equity and social inclusion, quality of life, and environmental sustainability. The smart city concept should be elaborated to solve all together SDGs in a synergistic mode like it was made implementing The United Smart Cities program initiated by The UN Economic Commission for Europe (UNECE). This is also the responsibility of political leaders at the local, regional, and global levels to ensure Smart Cities facilitate and don’t hamper the realization of the SDGs (Schwarz-Herion 2020).

The current Fourth Industrial Revolution with its fundamental transformation brings the opportunity to adapt and modernize governance models, to reduce social inequalities, and to commit to values-based leadership of emerging technologies (Schwab 2018). A smart city is the basic product of 4.0 technologies. Nowadays the digital infrastructure of a city becomes the center of society and all social processes of the urban population undergo the centripetal influence of the digital infrastructure of the city. The present time is characterized by the growing role of smart cities in the implementation of social policy. But Alvin Toffler in the 1980s predicted that in the future during the “information third wave” the center of society will be at home, at “digital cottage” (2004). The quarantine and self-isolation which the countries had to keep for several months can be called a hyperbolical forerunner of the future home-centered society model. It is obvious that the sociology of the city gradually transforms into the sociology of a smart city and will reflect the fundamental sociological changes with which society in the future will adopt a model of human-centered personal space or “Toffler’s digital cottage” (smart home).

It is important to recognize the large vector of sociological transformation as smart cities are just a transition phase. One of the features of a large vector of changes is the comprehensive and holistic care of the person as a unit of society. In this way, the most value for a human is health or wellness. The definition “sociology of smart city” doesn’t reflect enough the process of development toward the future per se. The “process” is more important in the digital world than “infrastructure” (for example, the process can include technology of augmented and virtual reality or the governance of the city relies on AI). The definition “sociology of smart city lifeway” is more relevant, and also the “sociology of smart citizen” reflects the trend of transition to home-centered (smart home) urban society. The term “sociology of smart citizen” seems to be the most perspective in front of the developing Fourth Industrial Revolution for which the main feature is cyber-physical systems, including the brain-computer interface. Although it is difficult today to predict which exact definition will be the most relevant in the future for a sociological study of a smart city in which city residents are connected to data and process management through a brain-computer interface, the “sociology of smart citizen” can be the right way on the next stage of technological evolution.

The vector of sociological transformation is leading to “atomization” of the world urban population, to the local level of smart city (functioning as interconnected “organism”), and to a smart home personal place. This raises the gap problem in achieving SDGs because of different approaches to constructing digital architectures for smart cities or smart homes in countries. Digital technologies are indispensable tools to eliminate gap within regions and cities with respect to the achievement of the SDGs (Johnson 2018; García Zaballos et al. 2019). At the beginning of the decisive decade for people and the planet while the window of opportunity is closing fast UN Secretary-General António Guterres said: “We must connect the dots across all that we do—as individuals, civic groups, corporations, municipalities and the Member States of the United Nations—and truly embrace the principles of inclusion and sustainability” (Global Sustainable Development Report 2019). The strategy of creating smart cities in this decade should bring each citizen closer to SDGs at the individual level, laying in the personal space the principles of sustainable development and wellness of personality. The approach we proposed is aimed at gap elimination on an individual level for each citizen in smart cities.

The evolution of human civilization development goals launched long before the Millennium Summit and relied on the pillar of the UN—**human rights**. Since The Universal Declaration of Human Rights adoption in 1948 and the Declaration on the Right to Development adoption in 1986 the accent on “rights” gradually has shifted to “**responsibility**.” In the UN's first Human Development Report (UNDP 1990) the definition of people’s well-being was done: “human development is a process of enlarging **people’s choices**,” which should lead to a long and healthy life, to acquire knowledge, and to have access to needed resources. The definition was addressed to Aristotle’s philosophical question of how political arrangement can facilitate people to achieve a “flourishing life.” The terms “sustained human development” and “global targets for human development” were appeared in this first report. During the following decades in the annual Human Development Reports the frequency of mention of the word “responsibility” raised and several terms appeared: social corporate responsibility, global responsibility for governance, responsibility for decision-making, country responsibility, collective responsibility, shared responsibility. The base value “the human rights” has intertwined with responsibility as “with rights come duties” (UNDP 2004).

As the Kyoto Protocol progresses the responsibility has spread to climate change: historic responsibility for climate and global warming, responsibility for environmental damage (UNDP 2007/8). Environment issues turned the human development tasks to ethical responsibility through a change in personal behavior. Then the requirements for responsibility strengthen and the term “**vulnerability**” appeared which reflected corruption and unresponsive state institutions (UNDP 2014). The implementation of development goals shifted to the decentralization of responsibility for governance, to local governments in a city, personal responsibility, responsible citizens, and responsibilities within households.

As the digital revolution has matured and the smart cities have been raising the lack of access to digital technologies began to be categorized as inequality and rights. The technological deprivation in slums as well as in traditional, “not smart” cities makes cities more vulnerable in front of twenty-first century challenges. Approximately 40% of the urban population by 2030 will live in slums, and inequality has become a major emerging urban challenge (United Nations Human Settlements Programme 2003; NATO Allied Command Transformation 2017). Global urban expansion without a stronger smart strategy confronts the 2030 Agenda and multiplies inequality and irresponsibility.

Mobile broadband, IoT, and AI being linked to every “smart” citizen can provide a personalized approach to everyone to keep **responsible choices** in daily life, and through this way to bring the implementation of SDGs to every family and home embracing the environment, health, opportunity and development (Fig. 6). On the other hand, it is crucial to set the SDGs in a superior place to compare with smart technologies, because digitalization processes today tend to act as “fire accelerants”, exacerbating existing non-sustainable trends such as the overuse of natural resources and growing social inequality in many countries (Fromhold-Eisebith et al. 2019), increasing vulnerability of human.

Over 40 years ago the founding father of human development, Professor Amartya Sen asked a simple question: equality of what? His answer was: of the things we care about to build the future we aspire to (UNDP 2019). The equal human development with personal responsible “sustainable” behavior is the red line passing through decades of maturation, awareness, and revision of views in global governance. Today the digital technology allows us due to common information space shaping the **values** the sustainable future needs with filling the concept of values by a clear understanding of rights, responsibility, vulnerability, and personal choice.

The main findings and added value of this study are the following.

1. We showed the capacity of API-sociology to investigate the Internet space stressed the importance of the new computational methods in studying the growing textual Big Data as the product of people’s interaction around the world that gives the opportunity to make the agile assessment of SDGs reflection in people global discussion and provides a fast reaction to any change of accents.
2. We revealed the combined global social topics having in common with the SDGs and with the high frequency of mention of morbidity that reflects the huge interest of the global Internet audience to this theme even before the appearance of the COVID-19 pandemic. In this way proposed the hybrid sociological and technological concept of a smart city with matched sociological and technological solutions, with the bionic solution for ensuring sufficient data governance, and with health policy incorporated into the sociology of a city.
3. We make the contribution to scientific discourse about the sociology of the city in a modern context related to “smart” digital technologies and SDGs implementation with a focus on personalized support for each “smart” citizen who seeks to live in a safe home-centered space, taking into account the importance of shaping the values in common information space, showing to people the dependence of health on the holistic implementation of SDGs, and introducing into the population the practice of responsible “sustainable” behavior.

## Supplementary Information

Below is the link to the electronic supplementary material.

Supplementary file1 (PDF 3934 KB)

## Data Availability

The study data is available from the investigators on reasonable request. Requests should be directed to the corresponding author by email.
