# Supplementary material for: Sociological modeling of smart city with the implementation of UN sustainable development goals
Source: Sustain Sci. 2021 Jan 3;16(2):581–99. doi: 10.1007/s11625-020-00889-5 (PMC7779083; doi:10.1007/s11625-020-00889-5)
Supplement: Supplementary file 1 — Supplementary file1 (PDF 3934 KB) [file 11625_2020_889_MOESM1_ESM.pdf]

Supplementary Information for

Sociological Modeling of Smart City with the implementation of UN Sustainable Development Goals

Olga Kolesnichenko, Lev Mazelis, Alexander Sotnik, Dariya Yakovleva, Sergey Amelkin, Ivan Grigorevsky, Yuriy Kolesnichenko

S.I.–Table 1 Cluster analysis k-means (iPython) of Morphological matrix – countries activity on the Internet

**Clusters**	**Countries**
Cluster 1	United States
Cluster 2	Canada, China, France, Germany, India, Japan, United Kingdom
Cluster 3	Australia, Belarus, Belgium, Chile, Colombia, Costa Rica, Denmark, Georgia, Indonesia, Israel, Italy, Kazakhstan, Latvia, Mexico, Morocco, Netherlands, Peru, Poland, Romania, Russia, South Africa, South Korea, Sri Lanka, Sweden, Switzerland, Taiwan, Turkey, Ukraine, Yemen
Cluster 4	Afghanistan, Albania, Armenia, Austria, Azerbaijan, Bahrain, Barbados, Benin, Bhutan, Brunei-Darussalam, Bulgaria, Cabo Verde, Cameroon, Côte d'Ivoire, Djibouti, Dominica, Dominican Republic, Egypt, El Salvador, Estonia, Finland, Guatemala, Guyana, Hong Kong, Hungary, Iran, Iraq, Ireland, Kosovo, Kyrgyzstan, Lebanon, Libya, Lithuania, Luxembourg, Madagascar, Malaysia, Malta, Mauritania, Mongolia, Montenegro, Namibia, New Zealand, Niger, Norway, Oman, Pakistan, Palestine, Paraguay, Portugal, Saint Lucia, Saudi Arabia, Serbia, Seychelles, Singapore, Slovakia, Slovenia, Swaziland, Syria, Tonga, Tunisia, Uzbekistan, Vanuatu, Vietnam

Note: Highest level of Internet activity and citing – Cluster 1; High level – Cluster 2; Middle level – Cluster 3; Low-Middle level – Cluster 4.

S.I.–Table 2 Morphological matrix for countries

**Units**	**Keywords**
Unit Information	*computer, cloud computing, software, artificial intelligence, smartphone, mobile phone, mobile app, dollar exchange rate, euro exchange rate*
Unit Energy & Material	*electric cars, solar panel, 3D printing, robotics, nuclear power plant, gas supplies, oil, oil price, drip irrigation, GMO, GMO harmful*
Unit Social inequality	*crisis, inflation, price increase, occupation, unemployment, dismissal, poverty*
Unit Stress load	*morbidity, mortality, narcotic, alcoholism, migrants, refugees, Muslims, terrorism, terrorist, violation, crime, war, casualties*
Unit Social profile	*revolution, demonstration, protest, strike, corruption, stability, prosperity, democracy, development, freedom, human rights, justice*

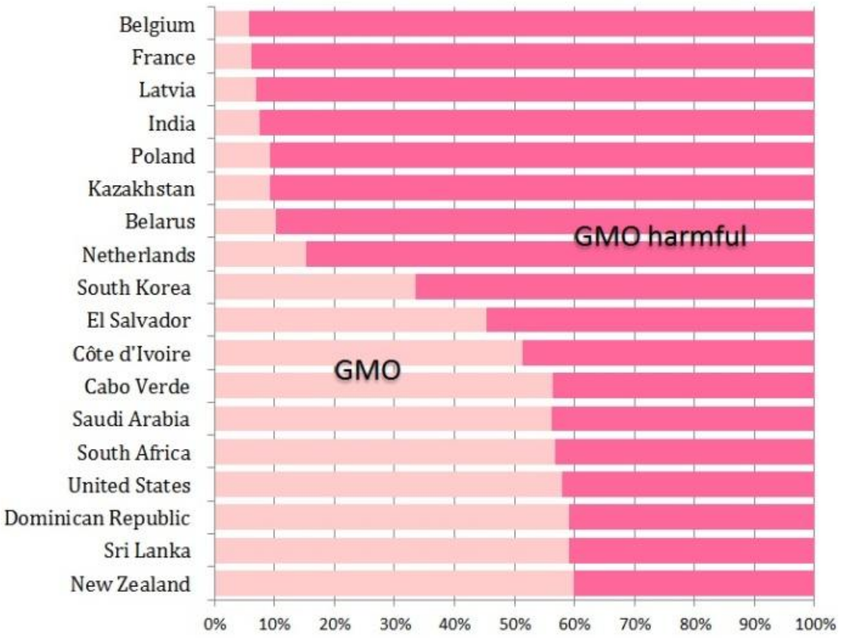

S.I.–Fig. 1 The percentage distribution of keywords *GMO* and *GMO harmful* on the Internet (Google API) related to some countries which have the prevalence of mention *GMO harmful*

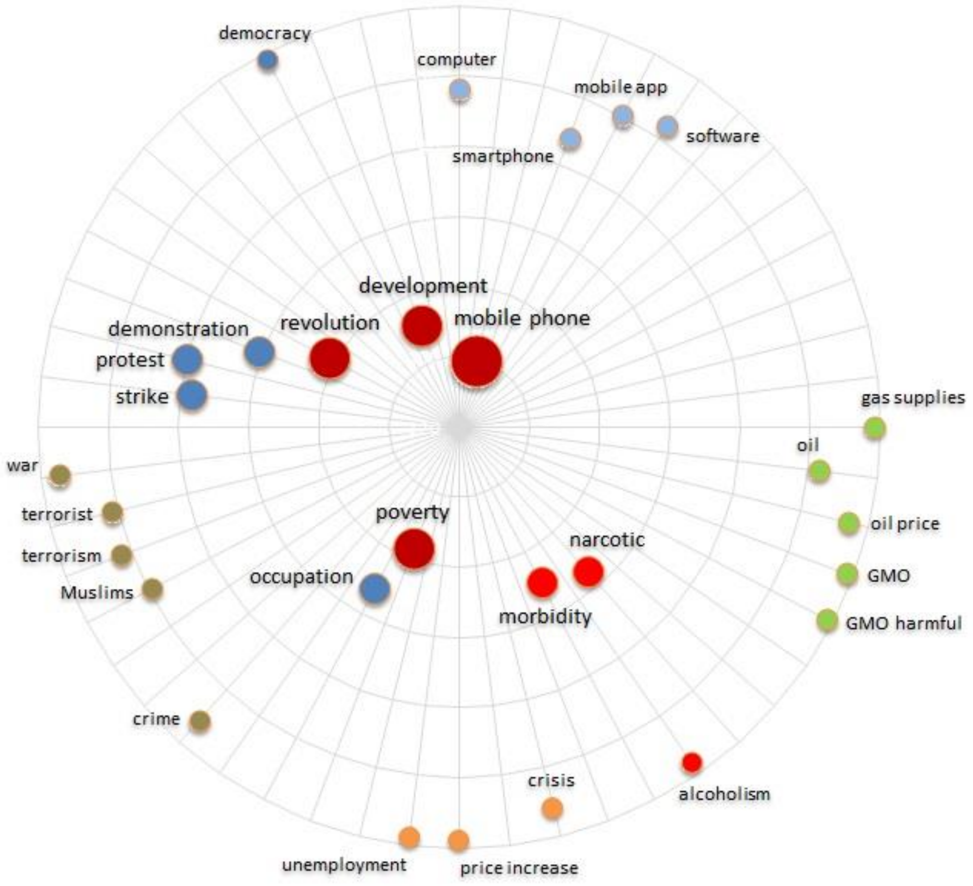

S.I.–Fig. 2 Global Trends Map, the distribution of selected keywords frequency on the Internet (Google API)

Note:
First published in:
Yakovleva DA, Kolesnichenko OYu, Mazelis LS, Grigorevsky IN, Kolesnichenko YuYu (2019) Api-sociology and Google global community: the live system of social transformation. Monitoring of Public Opinion: Economic and Social Changes 2:54–79. https://doi.org/10.14515/monitoring.2019.2.03

The closer to the center on the diagram, the more countries have a keyword with high-frequency. The size of the marker is scalable and also conditionally reflects the number of countries, which have a keyword with high-frequency. The keyword *revolution* can be associated with the Fourth Industrial Revolution.

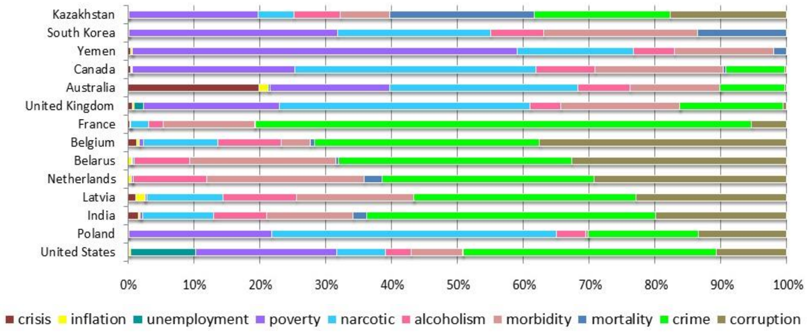

Group 1– emphasis on *crime*, *corruption*, and *mortality*

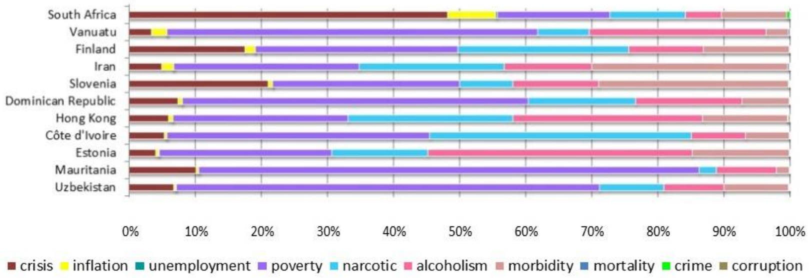

Group 2 – emphasis on *crisis*

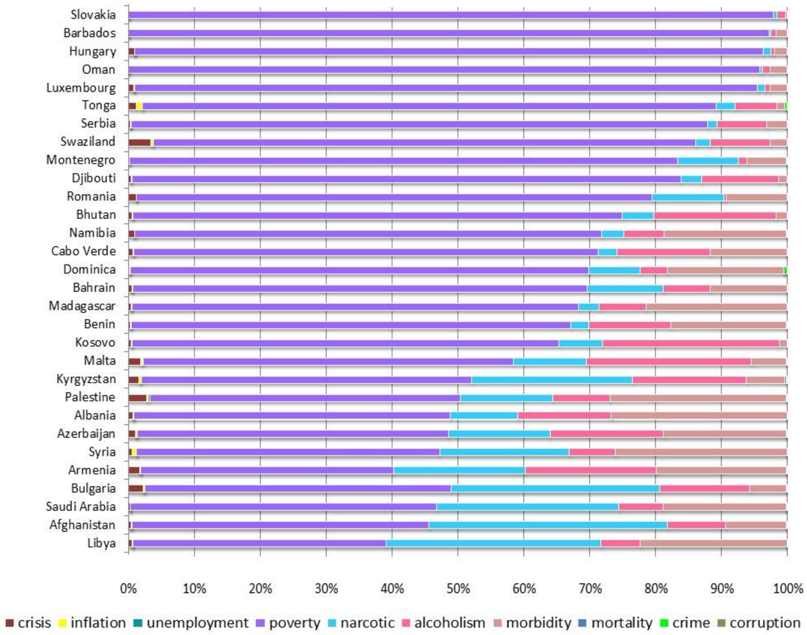

Group 3 – emphasis on *poverty*

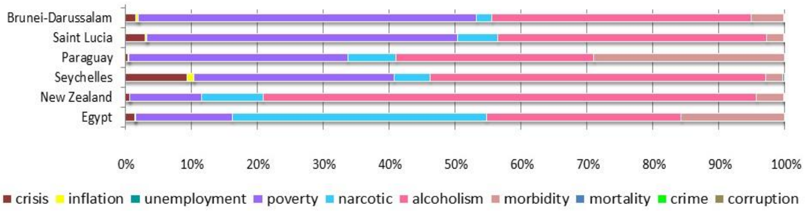

Group 4– emphasis on *alcoholism*

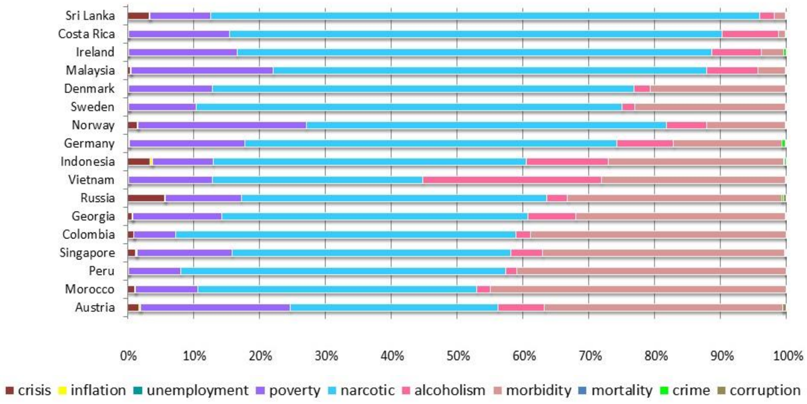

Group 5 – emphasis on *narcotic*

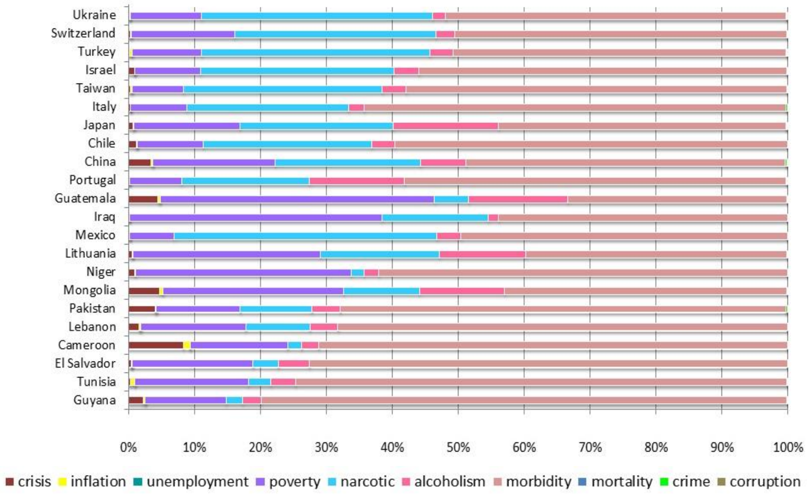

Group 6 – emphasis on *morbidity*

S.I.–Fig. 3 The percentage distribution of selected keywords of social issues on the Internet (Google API)

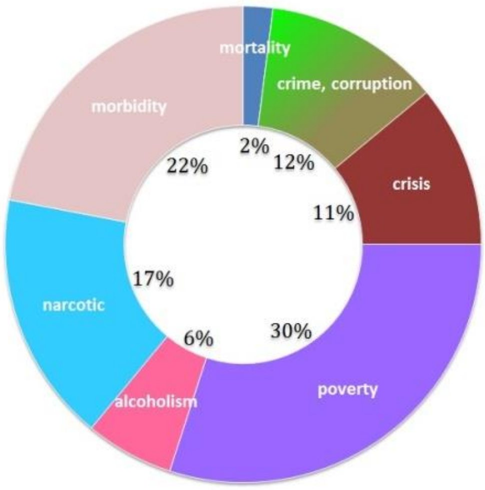

S.I.–Fig. 4 The percentage distribution of selected keywords accents within six groups of countries (see **S.I.–Fig. 3**) on the Internet (Google API)

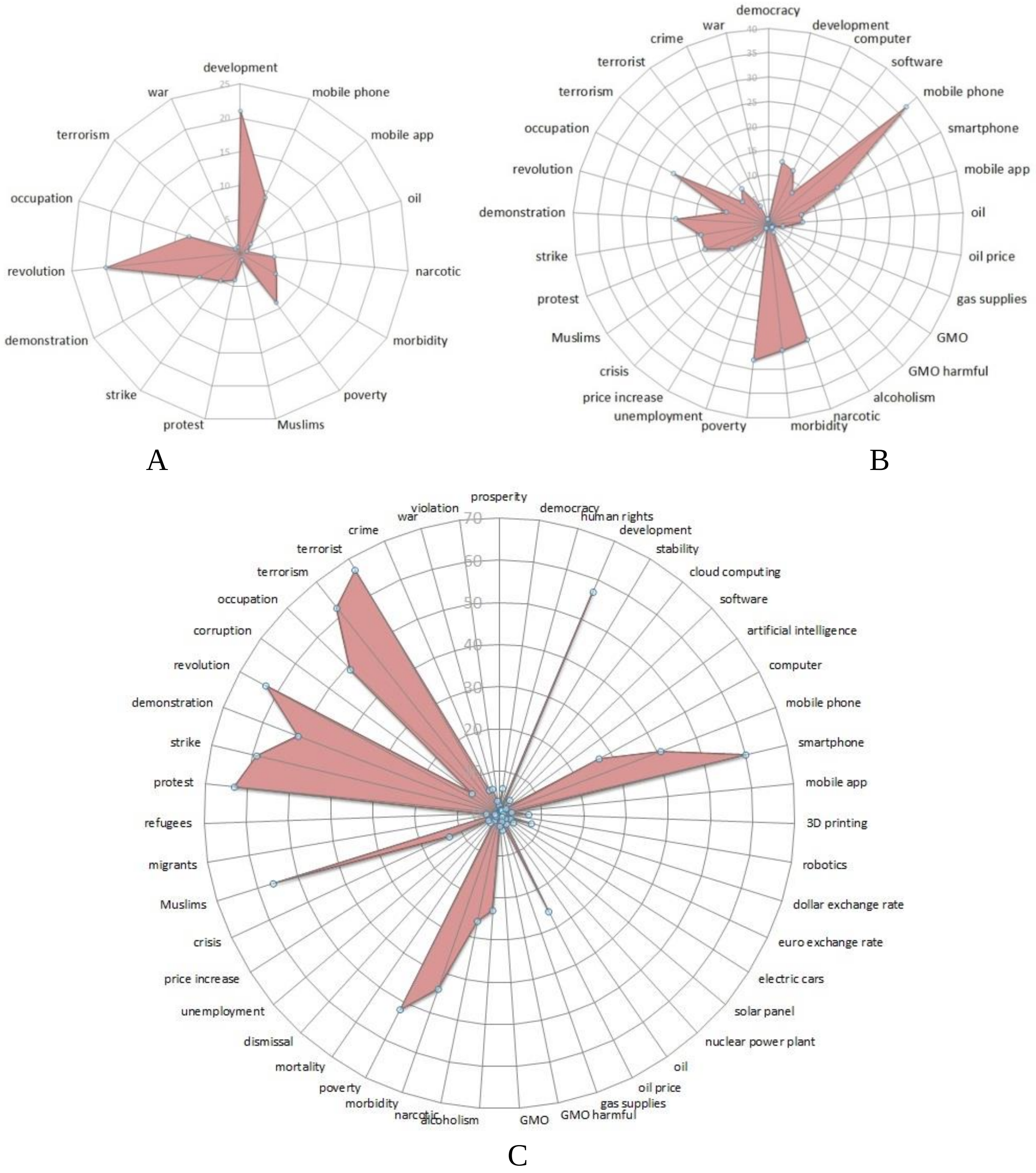

S.I.–Fig. 5 The leading global trends (the most expressed keywords patterns generalized for 100 countries)

Note:
The most expressed keyword for each country (A), the first level group of high-frequency mention of keywords (B), and the second level group (without keywords of the first level) of high-frequency mention of keywords, which are only noticeable after clearing data from the first level (C). Scale: number of countries which have high-frequency mention of keywords.

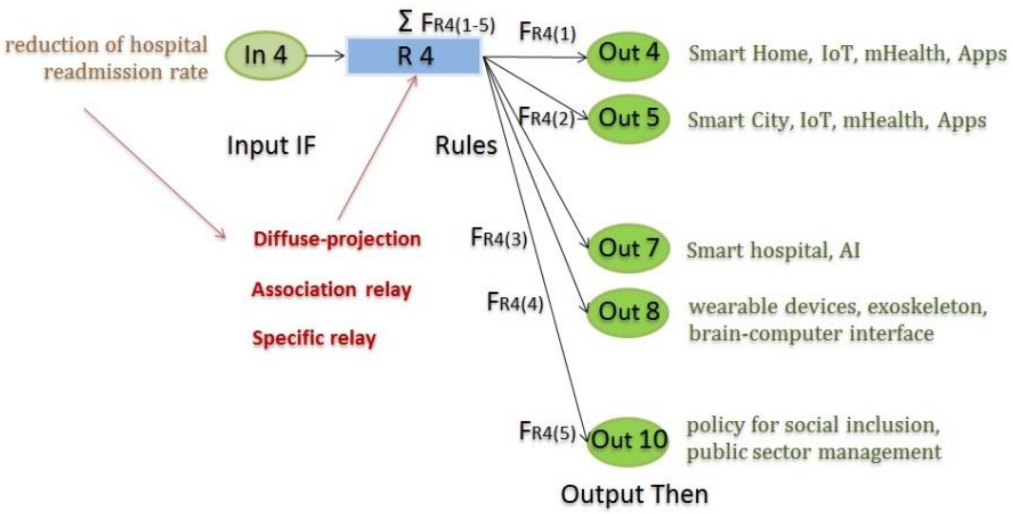

S.I.–Fig. 6 The example of the "one task – one rule" interconnection for several inferences within Logical AI scheme

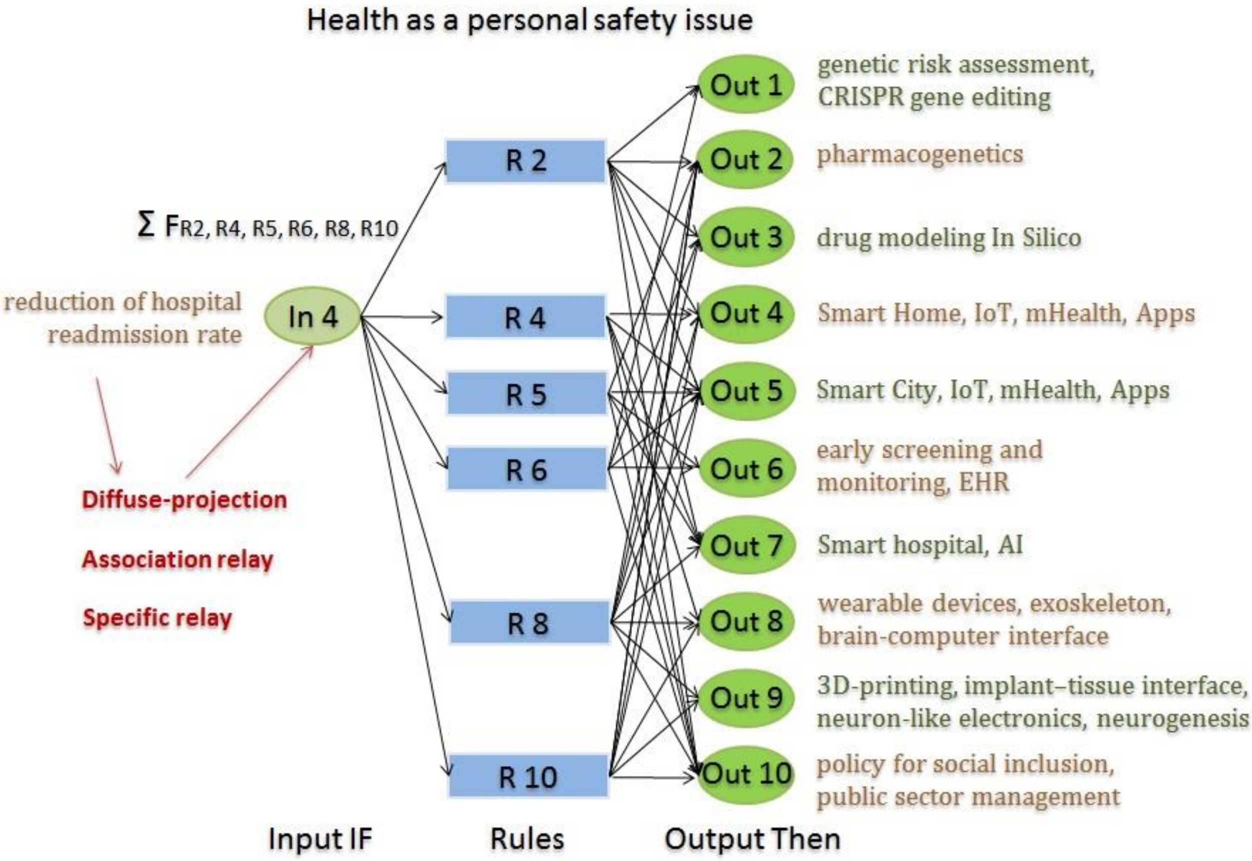

S.I.–Fig. 7 The example of the "one task – many rules" interconnection for several inferences within Logical AI scheme

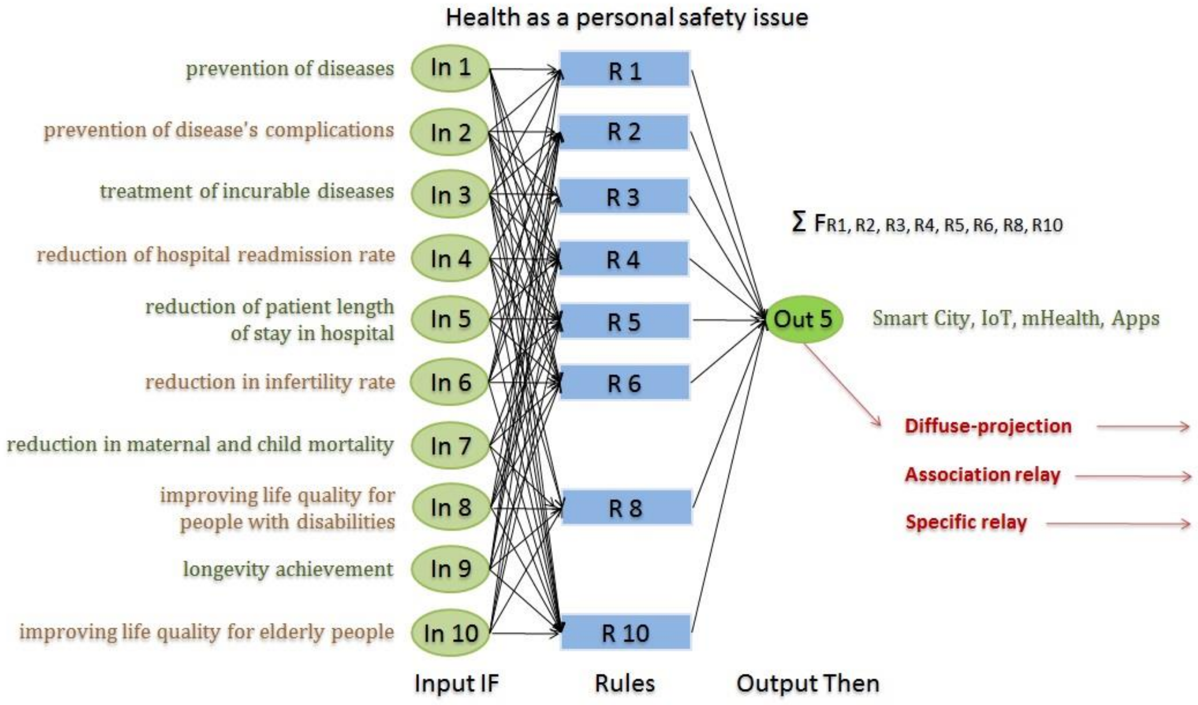

S.I.–Fig. 8 The example of the "several task – many rules" interconnection for one inference within Logical AI scheme

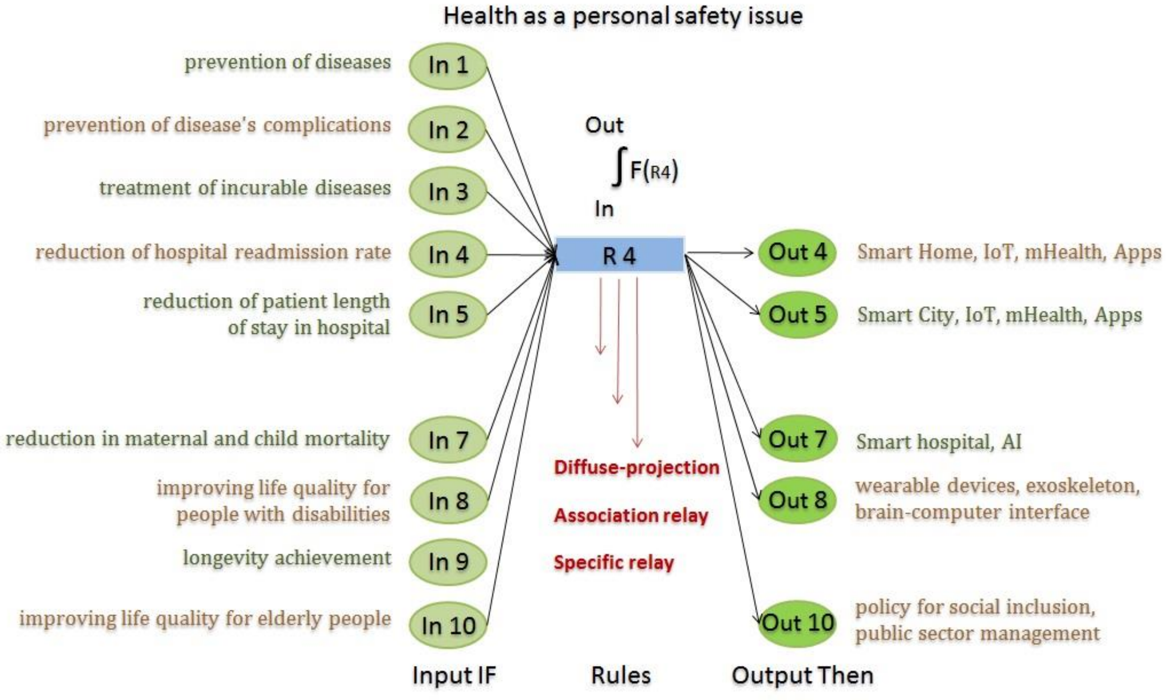

S.I.–Fig. 9 The example of the "many task – one rules" interconnection for several inferences within Logical AI scheme

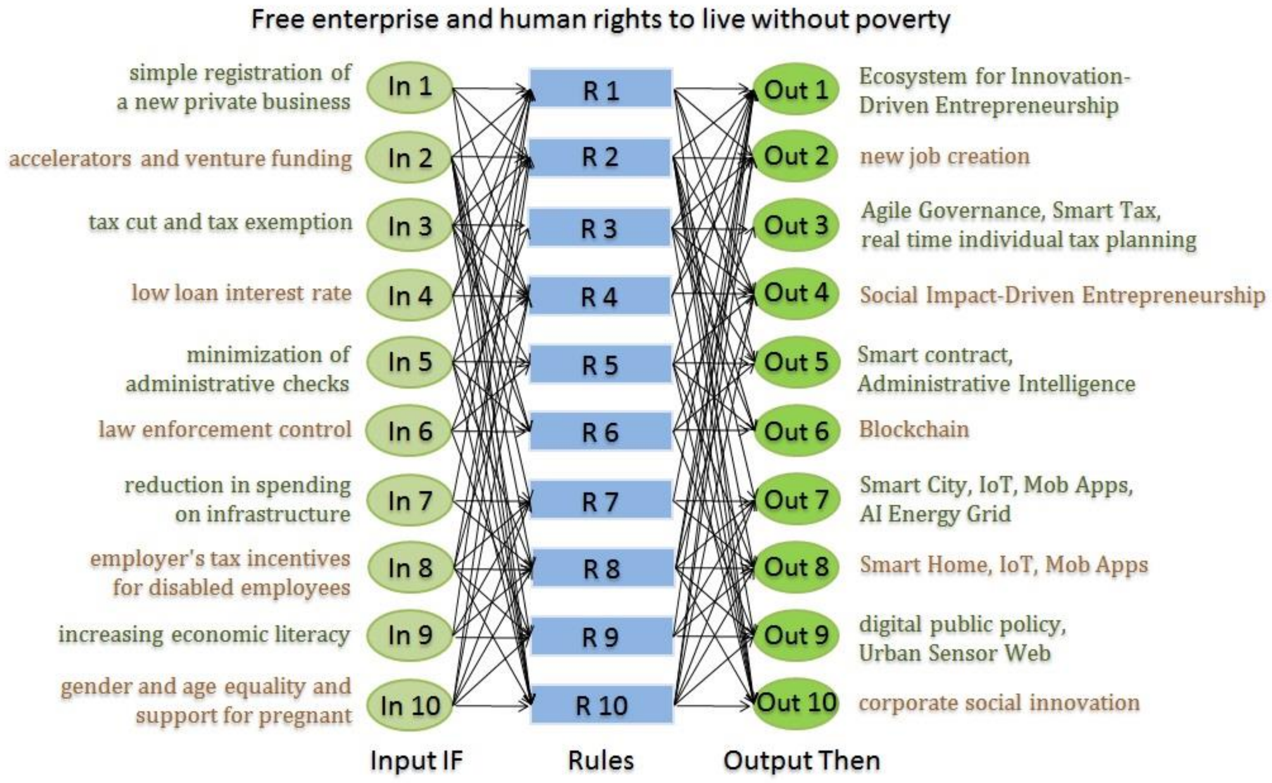

S.I.–Fig. 10 Logical AI scheme "Free enterprise and human rights to live without poverty" for a smart city

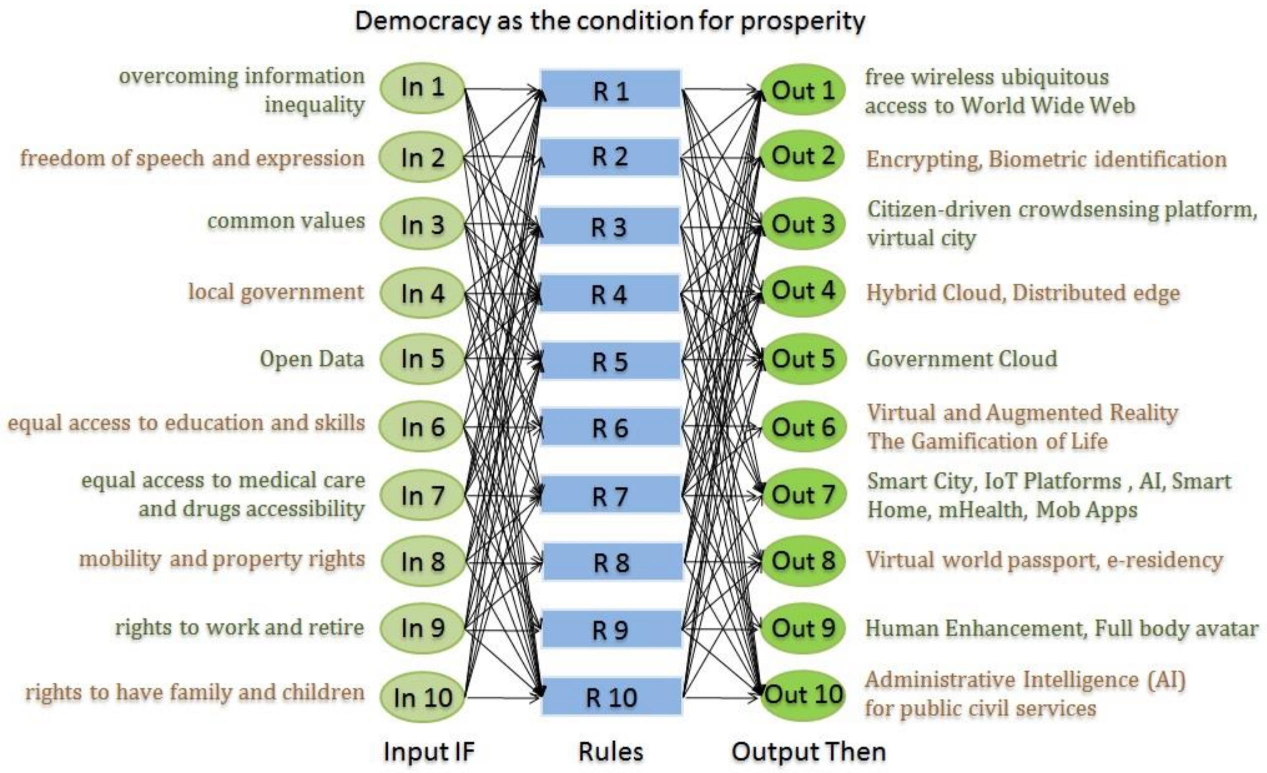

S.I.–Fig. 11 Logical AI scheme "Democracy as the condition for prosperity" for a smart city

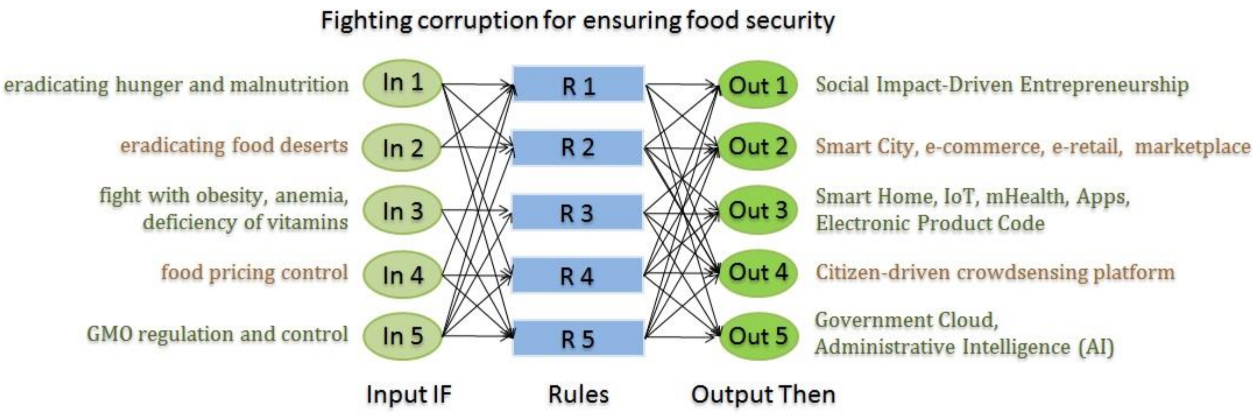

S.I.–Fig. 12 Logical AI scheme "Fighting corruption for ensuring food security" for a smart city

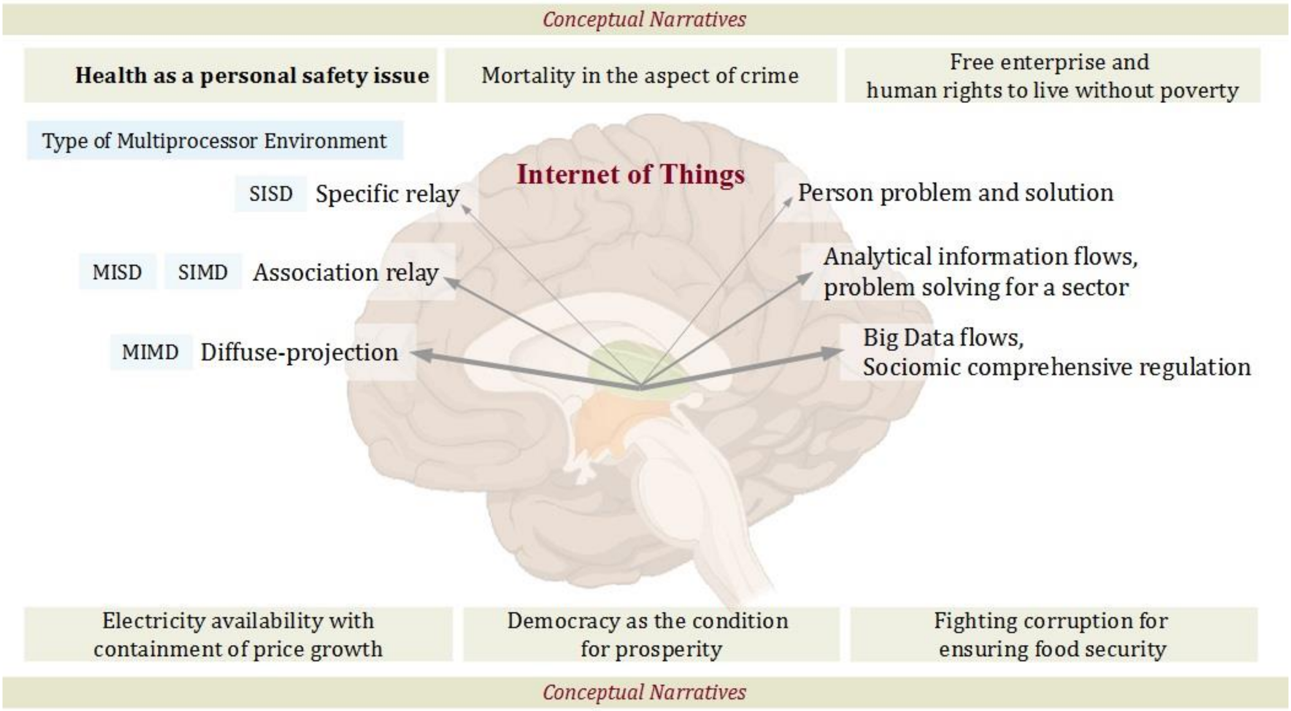

S.I.–Fig. 13 The juxtaposition of the three ways of redirected information flows by thalamic nuclei with three ways of information flows for data governance

Note:

SISD – Single Instruction, Single Data
MISD –Multiple Instruction, Single Data
SIMD – Single Instruction, Multiple Data
SIMD –Multiple Instruction, Multiple Data

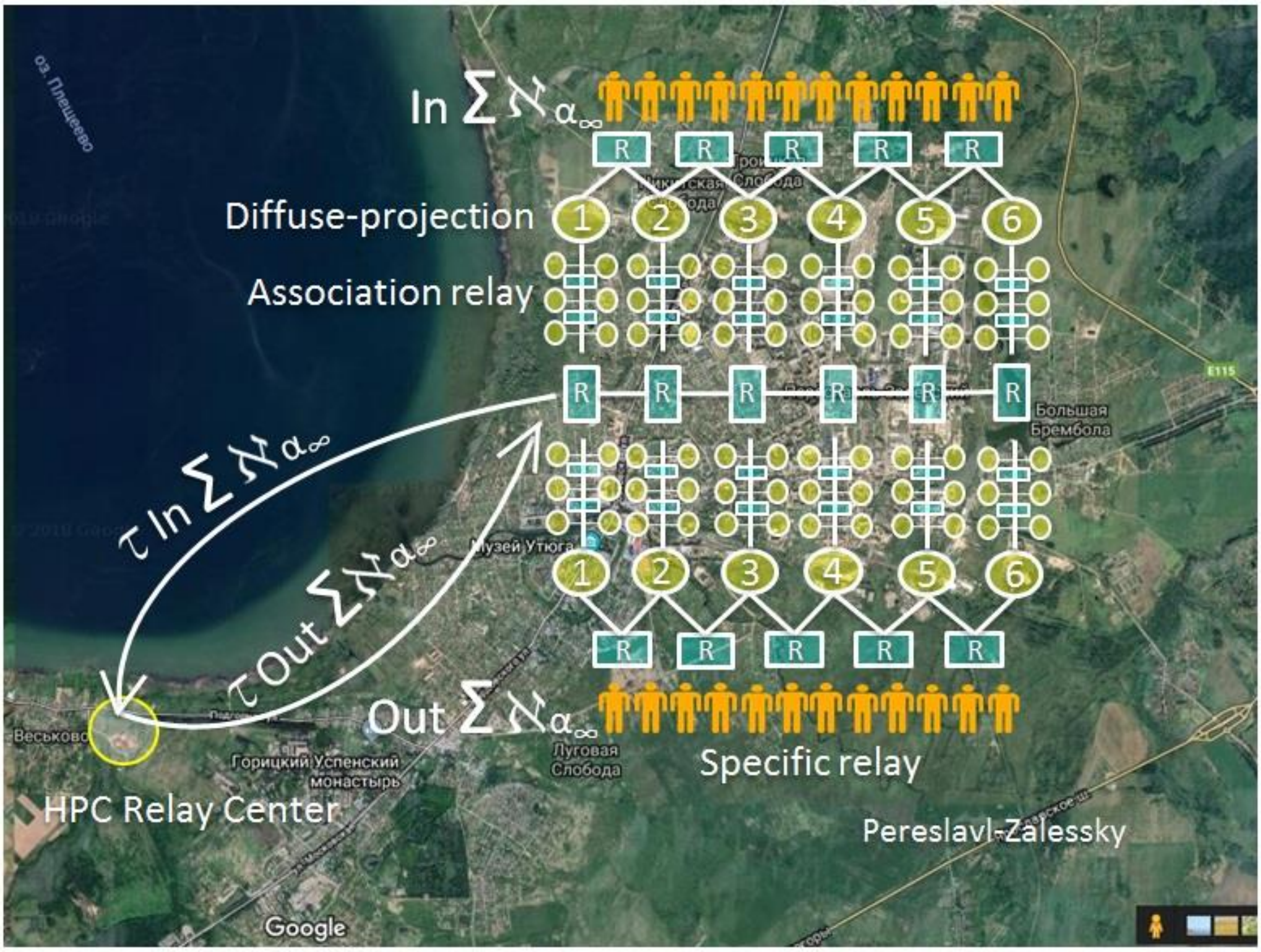

S.I.–Fig. 14 The digital architecture of a smart city – Pereslavl-Zalessky, Yaroslavl region, on the map

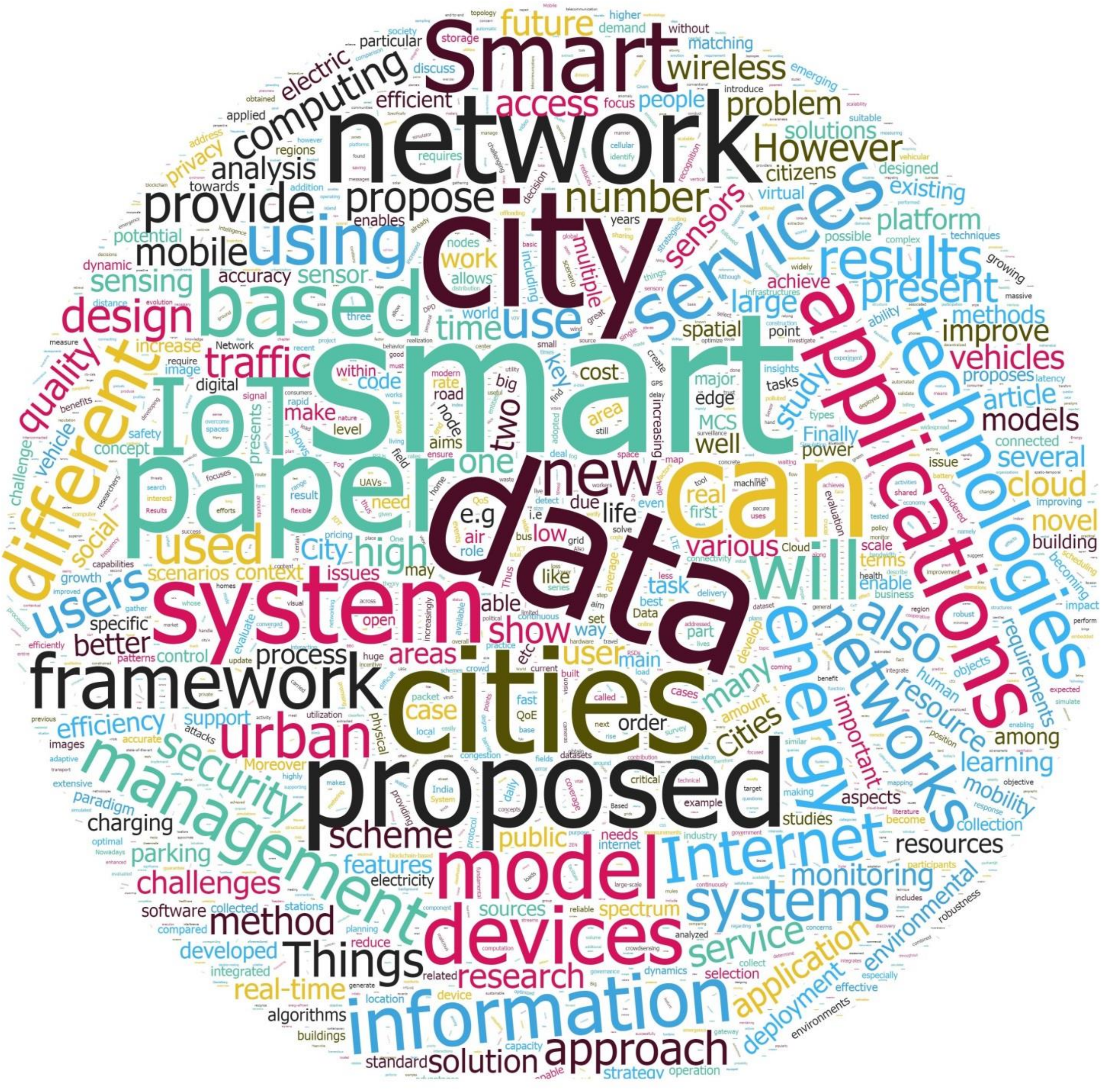

S.I.–Fig. 15 The word cloud (www.wordclouds.com, Java platform) based on query “Smart City”, IEEE Xplore Digital Library API

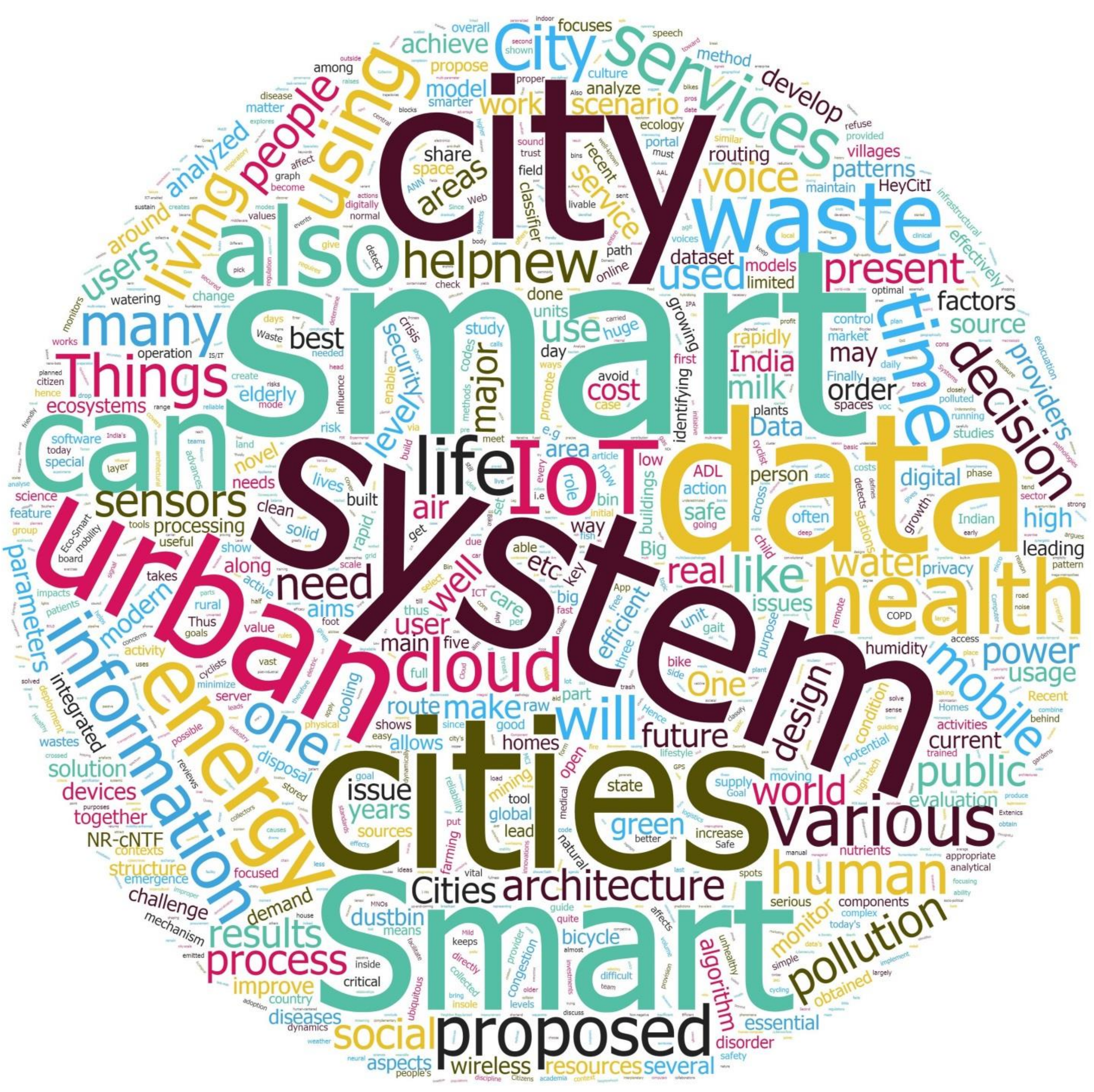

S.I.–Fig. 16 The word cloud (www.wordclouds.com, Java platform) based on query “Smart Healthy City”, IEEE Xplore Digital Library API

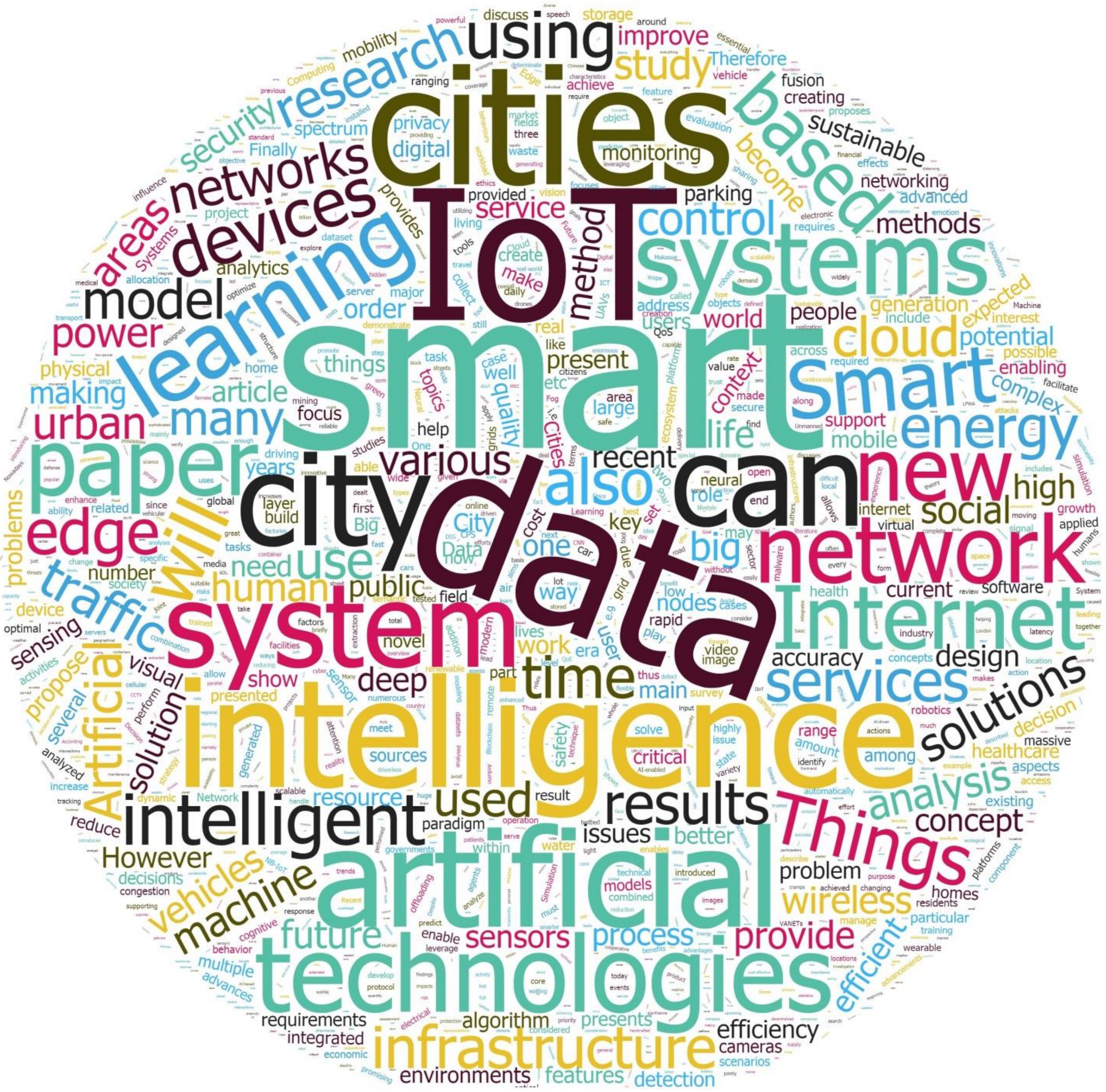

S.I.–Fig. 17 The word cloud (www.wordclouds.com, Java platform) based on query "Smart City + Artificial Intelligence", IEEE Xplore Digital Library API

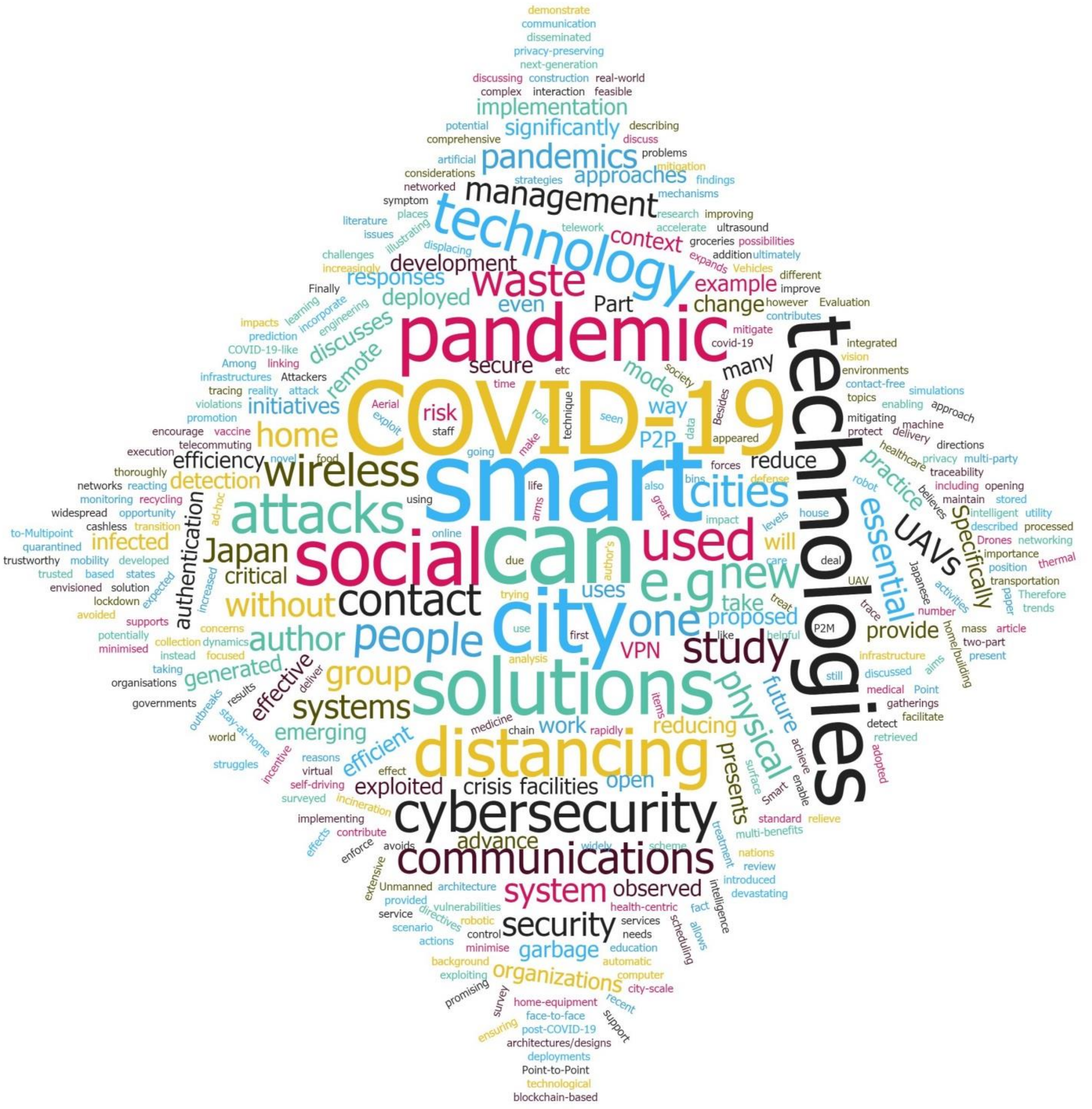

S.I.–Fig. 18 The word cloud (www.wordclouds.com, Java platform) based on query "Smart City + COVID", IEEE Xplore Digital Library API

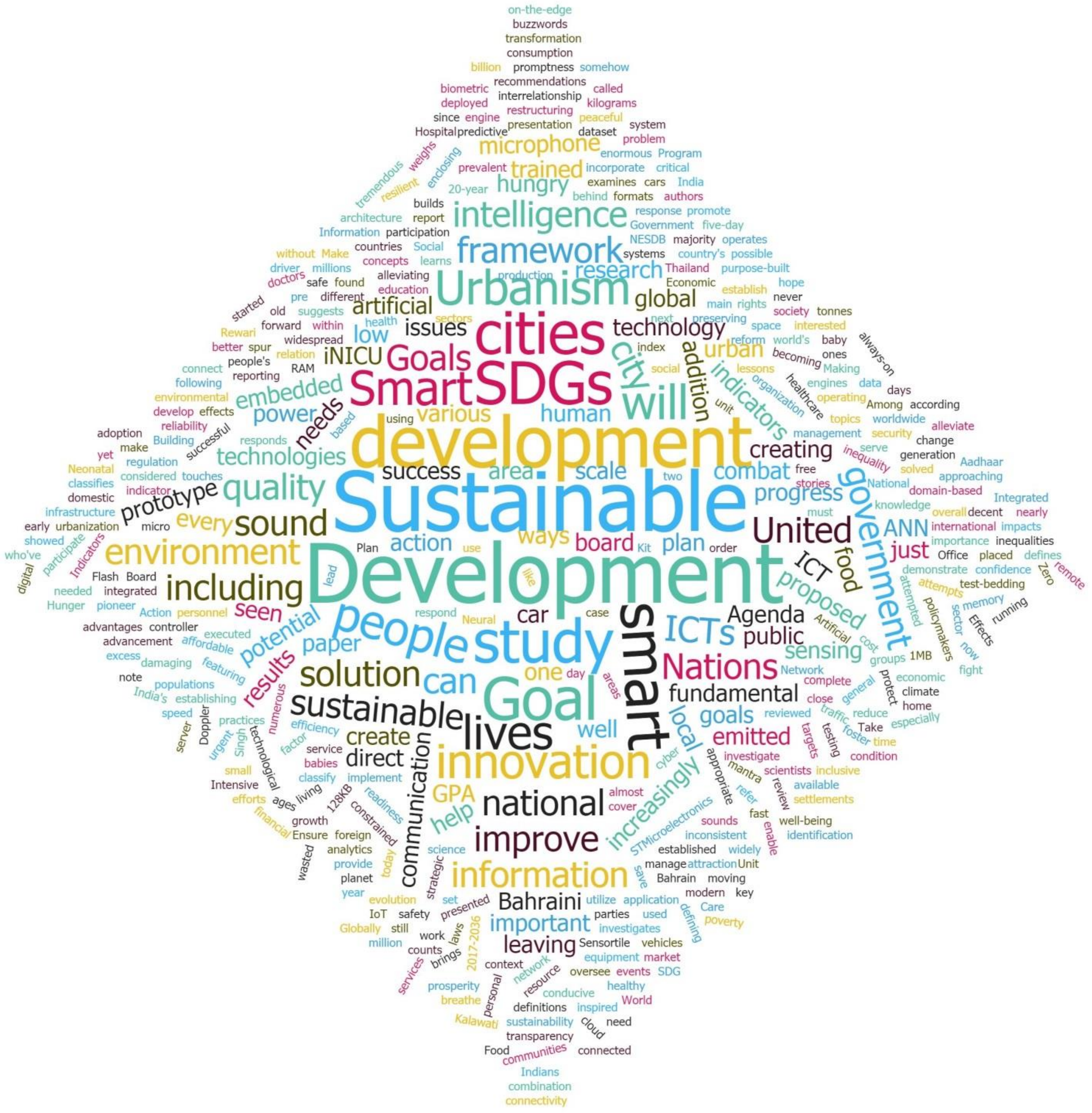

S.I.–Fig. 19 The word cloud (www.wordclouds.com, Java platform) based on query “Smart City + SDGs”, IEEE Xplore Digital Library API

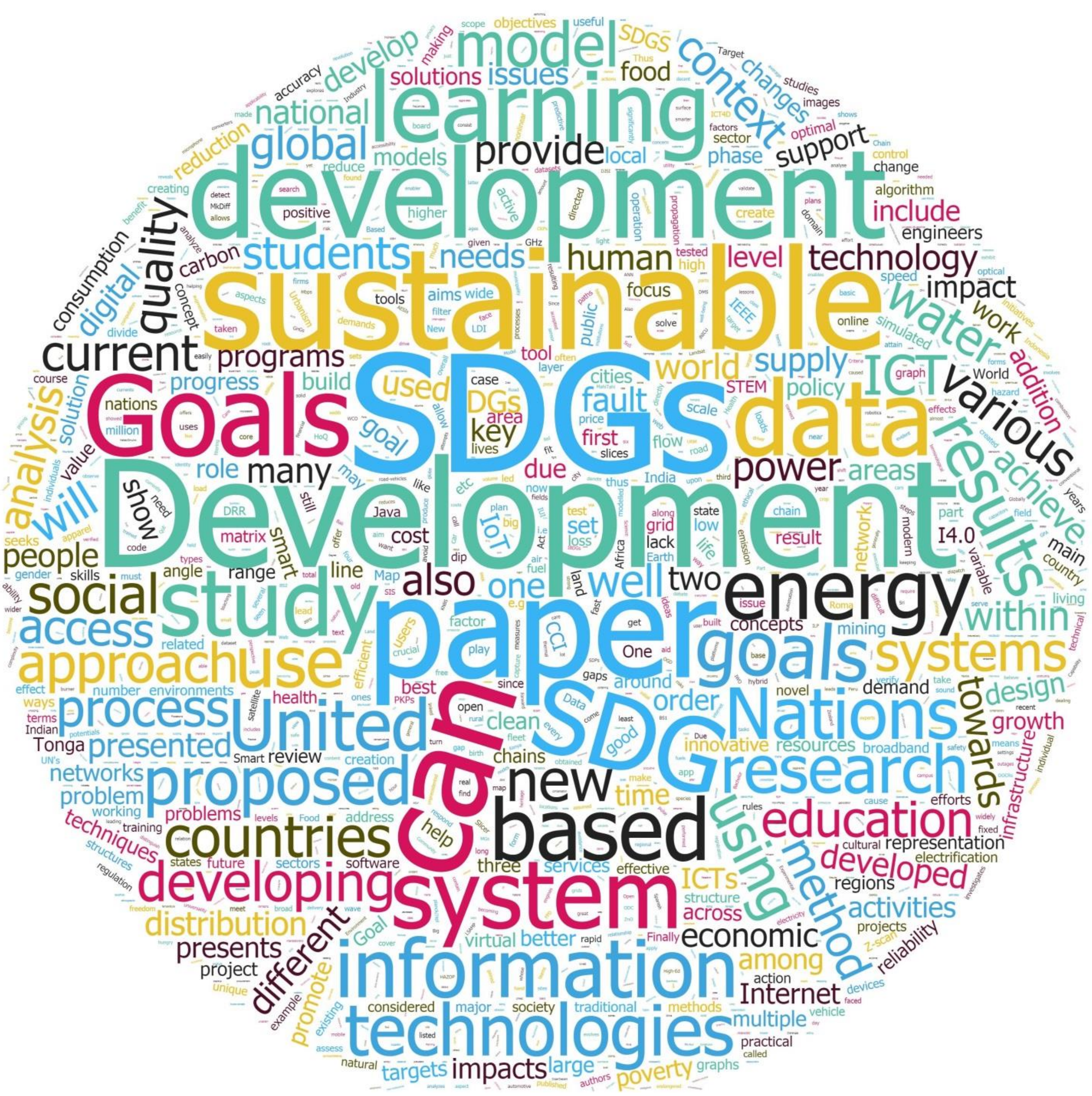

S.I.–Fig. 20 The word cloud (www.wordclouds.com, Java platform) based on query "SDGs", IEEE Xplore Digital Library API
